# TNFα-signal and cAMP-mediated signals oppositely regulate melanoma- associated ganglioside GD3 synthase gene in human melanocytes

**DOI:** 10.1038/s41598-019-51333-3

**Published:** 2019-10-14

**Authors:** Rika Takeuchi, Mariko Kambe, Maiko Miyata, Upul Jeyadevan, Orie Tajima, Koichi Furukawa, Keiko Furukawa

**Affiliations:** Department of Biomedical Sciences, Chubu University College of Life and Health Sciences, Matsumoto 1200, Kasugai, Aichi 487-8501 Japan

**Keywords:** Melanoma, Glycobiology

## Abstract

Analyses of expression and regulation of ganglioside synthases in melanocytes are important to understand roles of gangliosides in melanomagenesis. In this study, we analyzed the expression and regulatory mechanisms of glycosyltransferase genes responsible for ganglioside synthesis in normal melanocytes. We reported previously that culture supernatants of UVB-irradiated keratinocytes induced upregulation of ganglioside GD3 synthase gene in melanocytes, and mainly TNFα was responsible for it. Then, we found that elimination of dibutyryl cyclic AMP and IBMX from the medium also resulted in upregulation of the GD3 synthase gene. The addition of α-melanocyte-stimulating hormone which increases cAMP, to the medium led to a significant reduction in the GD3 synthase gene expression level, and a PKA inhibitor enhanced the GD3 synthase gene level. These results suggest that signals mediated via TNFα and cAMP oppositely regulate GD3 synthase gene expression in melanocytes. The results of an IKK inhibitor indicate the possibility that TNFα induces GD3 synthase gene expression via NF-κB signaling in melanocytes. When melanoma cells were treated by these factors, no fluctuation in the GD3 synthase gene expression level was observed, although an IKK inhibitor significantly suppressed it, suggesting that ganglioside synthase genes are regulated in distinct manners between melanocytes and melanomas.

## Introduction

**Figure 1:**
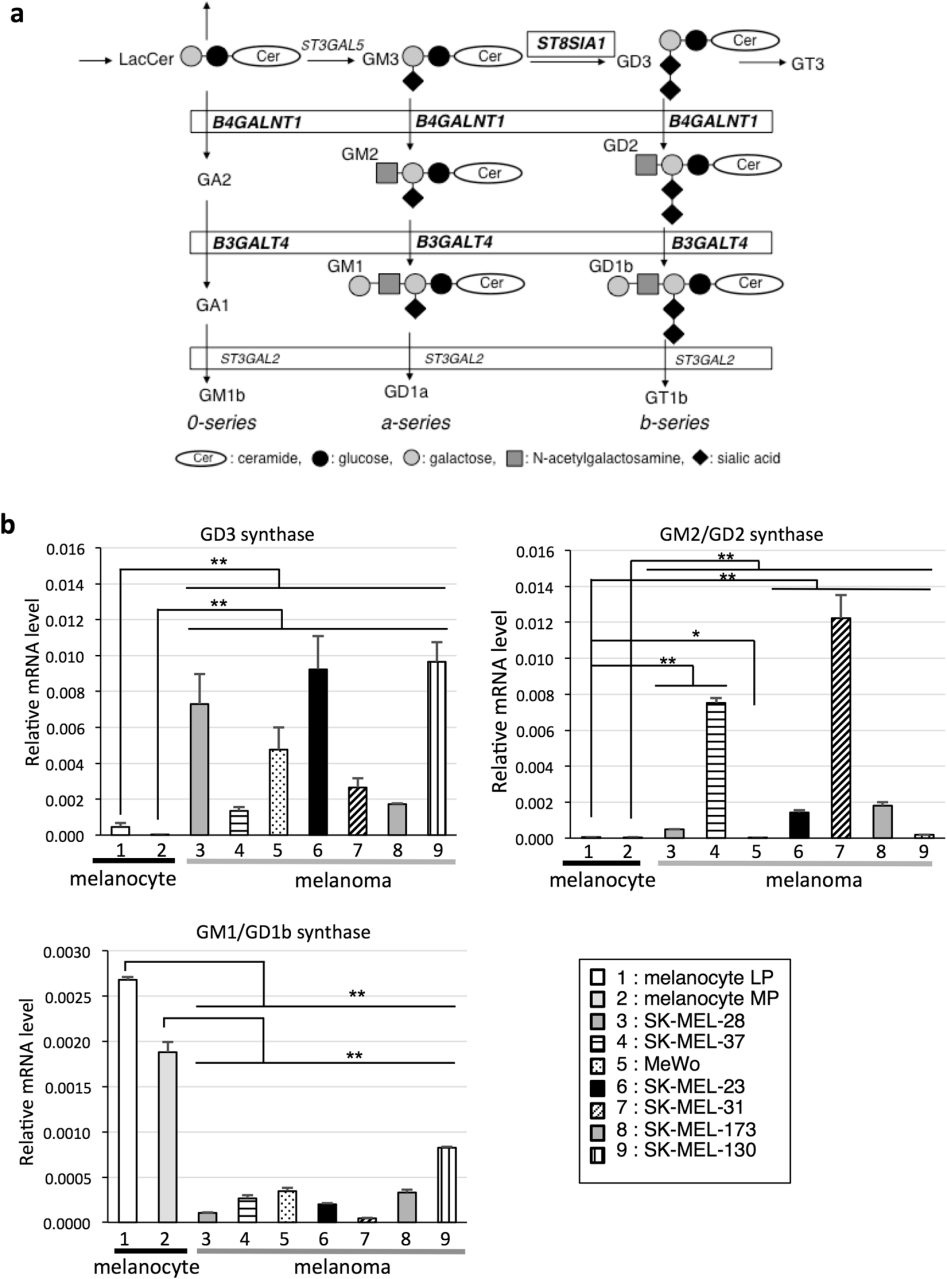
Synthetic pathway of sialic acid - containing glycosphingolipids, gangliosides and gene expression of glycosyltransferases in melanocytes and melanoma cell lines. (**a**) Glycosyltransferases genes for the synthesis of gangliosides are shown in italics. (**b**) Gene expression levels of glycosyltransferases in 2 melanocyte cells and 7 melanoma cell lines were analyzed. mRNA expressions of GD3 synthase, GM2/GD2 synthase, and GM1/GD1b synthase were analyzed by qRT-PCR in melanocytes (HEMn-LP (LP) and HEMn-MP (MP)) and melanoma lines (SK-MEL-28, SK-MEL-37, MeWo, SK-MEL-23, SK-MEL-31, SK-MEL-173, and SK-MEL-130). mRNA expression levels of these glycosyltransferases were normalized by those of human β-actin gene. Data represent means ± standard deviation (s.d.) (n = 4). Statistical analysis was performed by the two-tailed Student’s t-test (*P < 0.05; **P < 0.01).

Malignant melanomas develop from melanocytes, and retain various features of melanocytes such as pigmentation^1^. As for surface expression of glycosphingolipids on melanomas and melanocytes, there have been a number of studies with a focus on novel glycolipids that have emerged along with melanoma development^2^. First of all, ganglioside GD3 was reported to be a melanoma-associated ganglioside, since it was expressed almost exclusively in melanomas, but not in normal melanocytes^3–5^. GD3 is difficult to detect even on other kinds of normal cells and tissues except brain tissues at an early developmental stage and on activated T lymphocytes^6^. GD2 and GM2 were also reported to be melanoma-associated gangliosides, and to be self-antigens recognized by the immune system of patients^7,8^. On the other hand, it has long been considered that normal melanocytes do not express definite levels of gangliosides except GM3, the simplest structure of gangliosides^9^. Synthetic pathways of major gangliosides and glycosyltransferases involved in individual steps are shown in Fig. 1a.

However, as we reported recently, human melanocytes can be induced to express some gangliosides that have been considered as melanoma-associated structures based on treatment with inflammatory cytokines such as tumor necrosis factor α (TNFα) and interleukin-6 (IL-6)^10^. Interestingly, UVB-irradiated keratinocytes secreted these cytokines, leading to induction of the ganglioside GD3 synthase gene (*ST8SIA1*) in adjacent melanocytes, while the direct exposure of melanocytes to UVB did not result in induction of the gene. These results suggest that normal melanocytes can be induced to synthesize and express melanoma-associated gangliosides under inflammatory microenvironments^10^. These results also suggest roles of melanoma-associated gangliosides in melanomagenesis^11^, since ganglioside GD3 and/or GD2 produced via GD3 synthase is known to enhance malignant properties of malignant melanomas^12^, gliomas^13,14^, breast cancers^15^, small cell lung cancers^16^, and osteosarcomas^17^. In turn, melanocytes express only monosialyl ganglioside, which has been reported to suppress cancer phenotypes^18^. Recently, we also reported that individual gangliosides differentially affected cell phenotypes by using GM3-, GM2-, GM1-, GD3-, or GD2-expressing melanoma cell lines established by transfecting cDNA of glycosyltransferases. Consequently, cell growth and cell adhesion intensity of GD3-expressing cells and GD2-expressing cells increased. Cell invasion activity of GD3-expressing cells also increased, while cell adhesion of GD2-expressing cells were markedly strong^19^. Therefore, neo-expression of GD3 in melanocytes may bring about enhancement of cell growth and adhesion in melanocytes, leading to similar phenotypes as malignant melanomas. Actually, increased GD3 expression enhances the migration of melanocytes as reported by Otake *et al*.^20^.

There have been no comprehensive studies on the regulation of gangliosides or ganglioside synthase genes in melanocytes. In this study, we analyzed the regulation of various ganglioside synthase genes by TNFα- and cAMP-mediated signals, as a representative inflammatory cytokine and a typical melanocyte-differentiation signal^21^, respectively. Consequently, it was demonstrated that TNFα- and cAMP-mediated signals oppositely regulate the melanoma-associated GD3 synthase gene in human melanocytes, and that regulation of the gene in melanoma cells was different from that in melanocytes.

## Results

### GD3 synthase and GM2/GD2 synthase genes are scarcely expressed in melanocytes

We previously demonstrated that expression levels of GD3 synthase (*ST8SIA1*) and GM2/GD2 synthase (*B4GALNT1*) genes (Fig. 1a) were markedly high in melanoma lines compared with melanocytes. In contrast, GM1/GD1b synthase (*B3GALT4*) showed very low expression in melanoma lines. In addition to our previous results^10^, we analyzed one more line of melanocytes (HEMn-MP) and three more melanoma cell lines (SK-MEL-31, SK-MEL-173, and SK-MEL-130) to clearly show differences in the expression patterns of glycosyltransferase genes between melanocytes and melanomas. For the expression analysis, two normal human melanocyte lines, HEMn-LP (LP) and HEMn-MP (MP), cultured in the culture medium for melanocytes, F10-A medium (see Methods), and seven melanoma cell lines (SK-MEL-28, SK-MEL-37, MeWo, SK-MEL-23, SK-MEL-31, SK-MEL-173, and SK-MEL-130) were used. Expression levels of GD3 synthase, GM2/GD2 synthase, and GM1/GD1b synthase genes were analyzed by real-time qRT-RCR. Consequently, expression levels of GD3 synthase and GM2/GD2 synthase genes were clearly higher in melanomas than in melanocytes, while the expression level of the GM1/GD1b synthase gene was lower in melanomas (Fig. 1b), and these results were essentially the same as in our previous report^10^. From these results, distinct expression patterns of glycosyltransferase genes between melanocytes and melanomas were demonstrated.

### Both TNFα treatment and removal of dcAMP and IBMX result in enhancement of GD3 synthase gene expression levels

**Figure 2:**
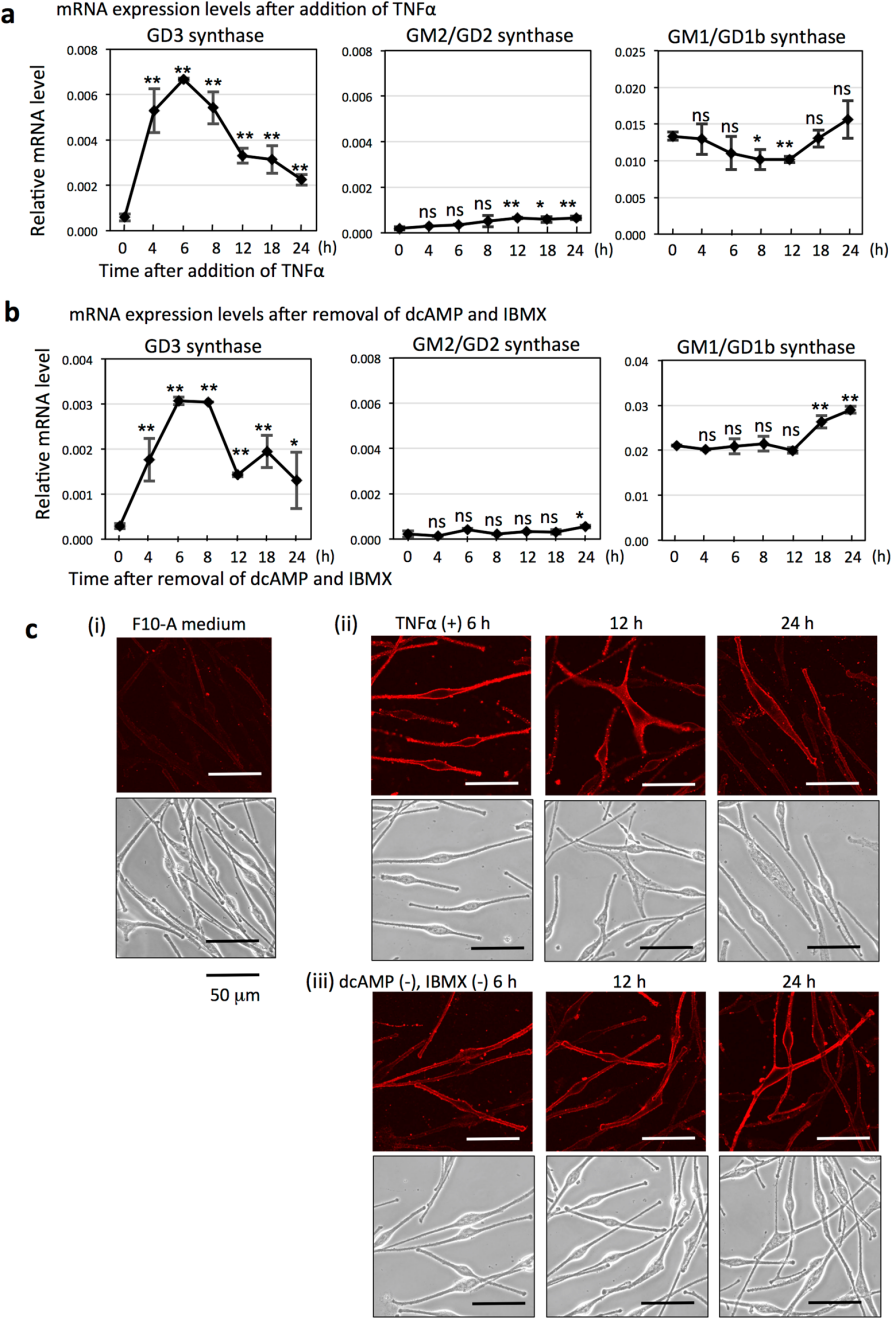
GD3 synthase gene upregulated by TNFα treatment or removal of dcAMP and IBMX from culture medium in melanocytes. (**a**) Time-course of mRNA expression levels of GD3 synthase, GM2/GD2 synthase, and GM1/GD1b synthase in melanocytes (LP) after adding TNFα to F10-A medium. Melanocytes were treated with TNFα (10 ng/mL) for 0–24 h. Then, mRNA expression levels were analyzed by qRT-PCR. mRNA expression levels of these glycosyltransferases were normalized by those of human GAPDH gene. Data represent means ± s.d. (n = 4). (**b**) Time-course of mRNA expression levels of glycosyltransferases in melanocytes (LP) after removing dcAMP (1 mM) and IBMX (0.1 mM) from F10-A medium. Melanocytes were cultured without dcAMP and IBMX for 0–24 h. Then, mRNA expression levels were analyzed by qRT-PCR. mRNA expression levels of these glycosyltransferases were normalized by those of human GAPDH gene. Data represent means ± s.d. (n = 4). Statistical analysis was performed by the two-tailed Student’s t-test (*P < 0.05; **P < 0.01). ns = not significant. (**c**) Immunofluorescence staining of GD3 of melanocytes (LP). Results on immunocytostaining with anti-GD3 antibody (R24) and Alexa Fluor 594-anti-mouse IgG were shown. Stained GD3 was observed using a confocal microscope (*upper*), and in bright field (*lower*). (i) Melanocytes were cultured in F10-A medium and GD3 expression was analyzed. (ii) GD3 expression was analyzed at 6, 12, and 24 h after the addition of TNFα (10 ng/mL) to F10-A medium. (iii) GD3 expression was analyzed at 6, 12, and 24 h after the removal of dcAMP and IBMX from F10-A medium (F10 medium).

Alteration of mRNA expression levels of GD3 synthase, GM2/GD2 synthase, and GM1/GD1b synthase after changes of culture conditions were analyzed by qRT-PCR in melanocytes. TNFα stimulation or removal of dcAMP and IBMX largely affected GD3 synthase gene expression levels in melanocytes. IBMX is an inhibitor of cAMP phosphodiesterase, resulting in accumulation of cAMP. Gene expression of GD3 synthase was increased provisionally, and mRNA levels reached their peak at 6~8 h after adding TNFα (10 ng/mL), but mRNA expression levels of GM2/GD2 synthase and GM1/GD1b synthase were mostly stable (Fig. 2a). GD3 synthase gene expression also reached the peak at 6~8 h after removing dcAMP (1 mM) and IBMX (0.1 mM) from F10-A medium, but mRNA expression levels of GM2/GD2 synthase and GM1/GD1b synthase were mostly stable (Fig. 2b). Western immunoblottings with anti-GD3 synthase, anti-GM2/GD2 synthase, and anti-GM1/GD1b synthase were performed using cell lysates prepared from melanocytes after adding TNFα (10 ng/mL) or removing dcAMP and IBMX. In the result, GD3 synthase in melanocytes was increased after adding TNFα (10 ng/mL) or removing dcAMP and IBMX. GM2/GD2 synthase was not detected after any treatment. GM1/GD1b synthase was increased after these treatments, although mRNA levels were not increased clearly (Supplementary Fig. S1). Results of immunofluorescence staining of GD3 on melanocytes are shown in Fig. 2c. GD3 was scarcely expressed in all cells during the culture in F10-A medium containing dcAMP and IBMX (Fig. 2c(i)). When melanocytes were cultured with TNFα (10 ng/mL) for 6, 12, or 24 h, GD3 was highly expressed on the cell membrane and at the tip of cell protrusion (Fig. 2c(ii)). When melanocytes were cultured in the medium without dcAMP and IBMX for 6, 12, or 24 h, expression of GD3 was highly induced in cells (Fig. 2c(iii)). Low magnification images of immunofluorescence staining of GD3 are shown, and quantification of fluorescent detected in each cell is shown in Supplementary Fig. S2.

### Factors enhancing cAMP-dependent protein kinase (PKA) signaling pathway suppress expression of GD3 synthase gene in melanocytes

**Figure 3:**
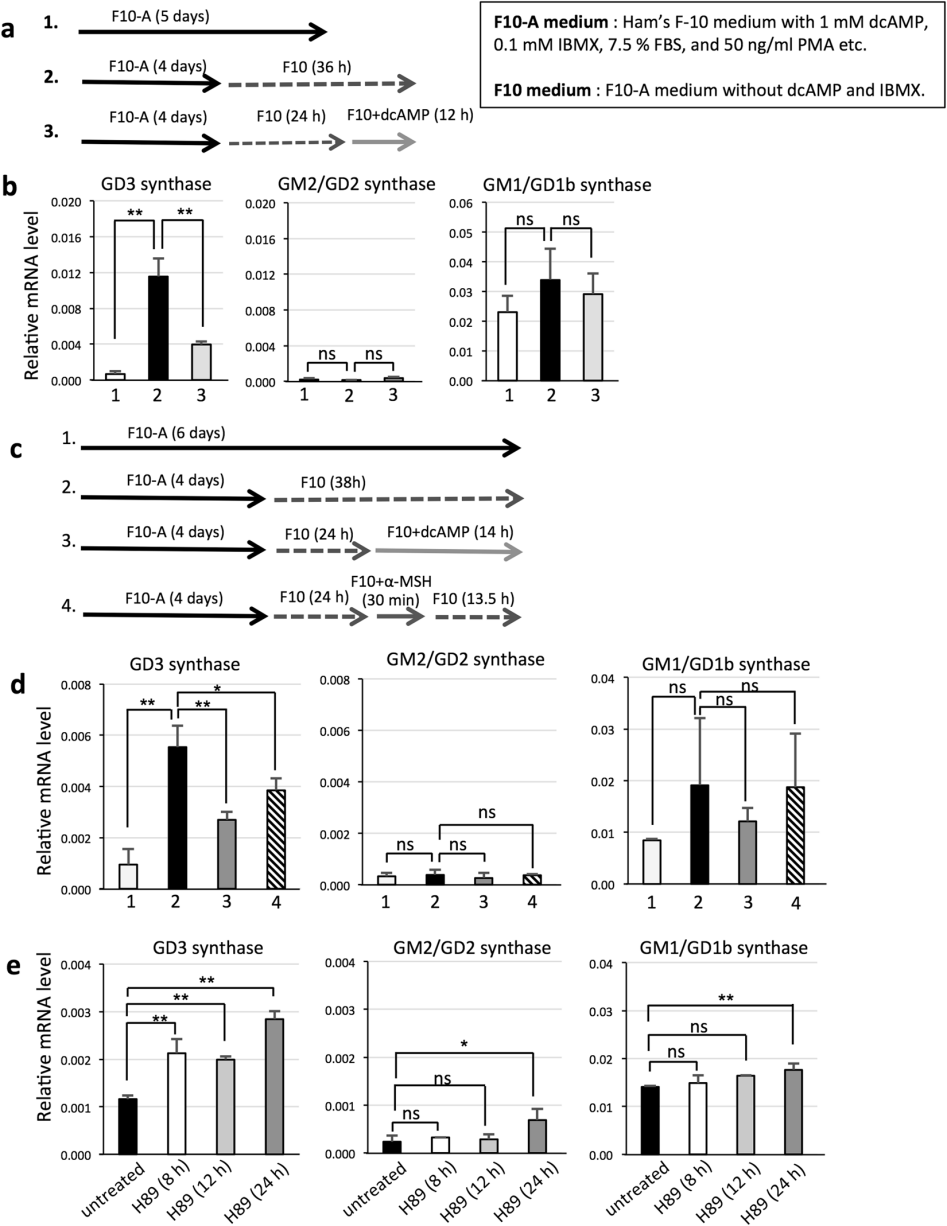
cAMP signal suppresses expression of GD3 synthase gene in melanocytes. (**a**) Melanocytes (LP) were cultured according to protocols 1~3. 1. Melanocytes were cultured in F10-A medium. 2. F10-A medium was exchanged with F10-A medium without dcAMP and IBMX (F10 medium) on day 5. 3. F10-A medium was exchanged with F10 medium on day 5, then dcAMP (1 mM) was added on day 6 and cultured for 12 h. (**b**) Melanocytes were cultured as shown in (a), and mRNA expression levels of GD3 synthase, GM2/GD2 synthase, and GM1/GD1b synthase were analyzed by qRT-PCR. mRNA expression levels of these glycosyltransferases were normalized by those of human GAPDH gene. Data represent means ± s.d. (n = 4). (**c**) Melanocytes (LP) were cultured according to protocols 1~4. 1. Melanocytes were cultured in F10-A medium. 2. F10-A medium was exchanged with F10 medium on day 5. 3. F10-A medium was exchanged with F10 medium on day 5, and dcAMP (1 mM) was added on day 6. 4. F10-A medium was exchanged with F10 medium on day 5. On day 6, α-MSH (100 nM) was added for 30 min, then medium was exchanged with F10 medium again. (**d**) Inhibitory effects of α-MSH on expression of the GD3 synthase gene in melanocytes. Melanocytes were cultured as shown in (**c**), and mRNA expression levels of GD3 synthase, GM2/GD2 synthase, and GM1/GD1b synthase were analyzed by qRT-PCR. mRNA expression levels of these glycosyltransferases were normalized by those of human GAPDH gene. Data represent means ± s.d. (n = 4). (**e**) Enhancing effects of a PKA inhibitor, H89, on expression of the GD3 synthase gene in melanocytes (LP). Melanocytes were cultured in F10-A medium for 4 days, and then an inhibitor of PKA, H89, was added. After treatment of melanocytes with H89 (10 μM) for 0, 8, 12, or 24 h, mRNA expression levels of GD3 synthase, GM2/GD2 synthase, and GM1/GD1b synthase were analyzed by qRT-PCR. mRNA expression levels of these glycosyltransferases were normalized by those of human GAPDH gene. Data represent means ± s.d. (n = 4). Statistical analysis was performed by two-tailed Student’s t-test (*P < 0.05; **P < 0.01). ns = not significant.

Melanocytes were cultured in F10-A medium for 4 days, and then, we prepared melanocytes cultured under various conditions as shown in Fig. 3a. We examined whether re-addtion of dcAMP affects the elevated expression level of the GD3 synthase gene in melanocytes. Consequently, GD3 synthase gene expression was increased after removing dcAMP and IBMX from F10-A medium as indicated in Fig. 3, and was suppressed at 12 h after the re-addition of dcAMP (1 mM) (Fig. 3b). However, mRNA expression levels of GM2/GD2 synthase and GM1/GD1b synthase were mostly constant.

A melanocyte-stimulating hormone, α-MSH, activates adenyl cyclase and promotes the conversion of ATP to cAMP, leading to activation of the cAMP-PKA signaling pathway. Melanocytes were cultured in F10-A medium for 4 days, and then we prepared melanocytes cultured under various conditions as shown in Fig. 3c. GD3 synthase gene expression was increased after removing dcAMP and IBMX from the culture medium, and was suppressed by adding either dcAMP (1 mM) or α-MSH (100 nM) (Fig. 3d). However, mRNA expression levels of GM2/GD2 synthase and GM1/GD1b synthase were mostly constant. Since H89 is a known inhibitor of PKA, we examined the effects of H89 on PKA signaling. Melanocytes were cultured in F10-A medium for 4 days, and then melanocytes were cultured with H89 (10 μM) for 8, 12, or 24 h. GD3 synthase gene expression was increased by adding H89 to culture medium, and the effect of H89 addition was maintained for at least 24 h (Fig. 3e). mRNA expression levels of GM2/GD2 synthase and GM1/GD1b synthase showed almost no change. α-MSH or H89 was dissolved in DMSO and added to each culture medium (100 nM α-MSH or 10 μM H89 as a final concentration). The solvent, DMSO showed no effect on relative mRNA levels (Supplementary Fig. S3). To confirm the inhibition effect of H89, melanocytes were cultured without dcAMP and IBMX for 17 h and cultured with or without H89 (10 μM) for 1 h, then stimulated with dcAMP (1 mM). Finally, phosphorylation of CREB was examined by western immunoblotting, showing that phosphorylation levels of CREB were decreased by treatment with H89 (Supplementary Fig. S4).

We confirmed GD3 synthase gene expression in other melanocytes: HEMn-MP also showed increased expression after removing dcAMP and IBMX or adding H89, i.e., by the inhibition of PKA signaling (Supplementary Fig. S5).

### Expression level of GD3 synthase gene increases synergistically with the addition of TNFα and inhibition of PKA signaling

**Figure 4:**
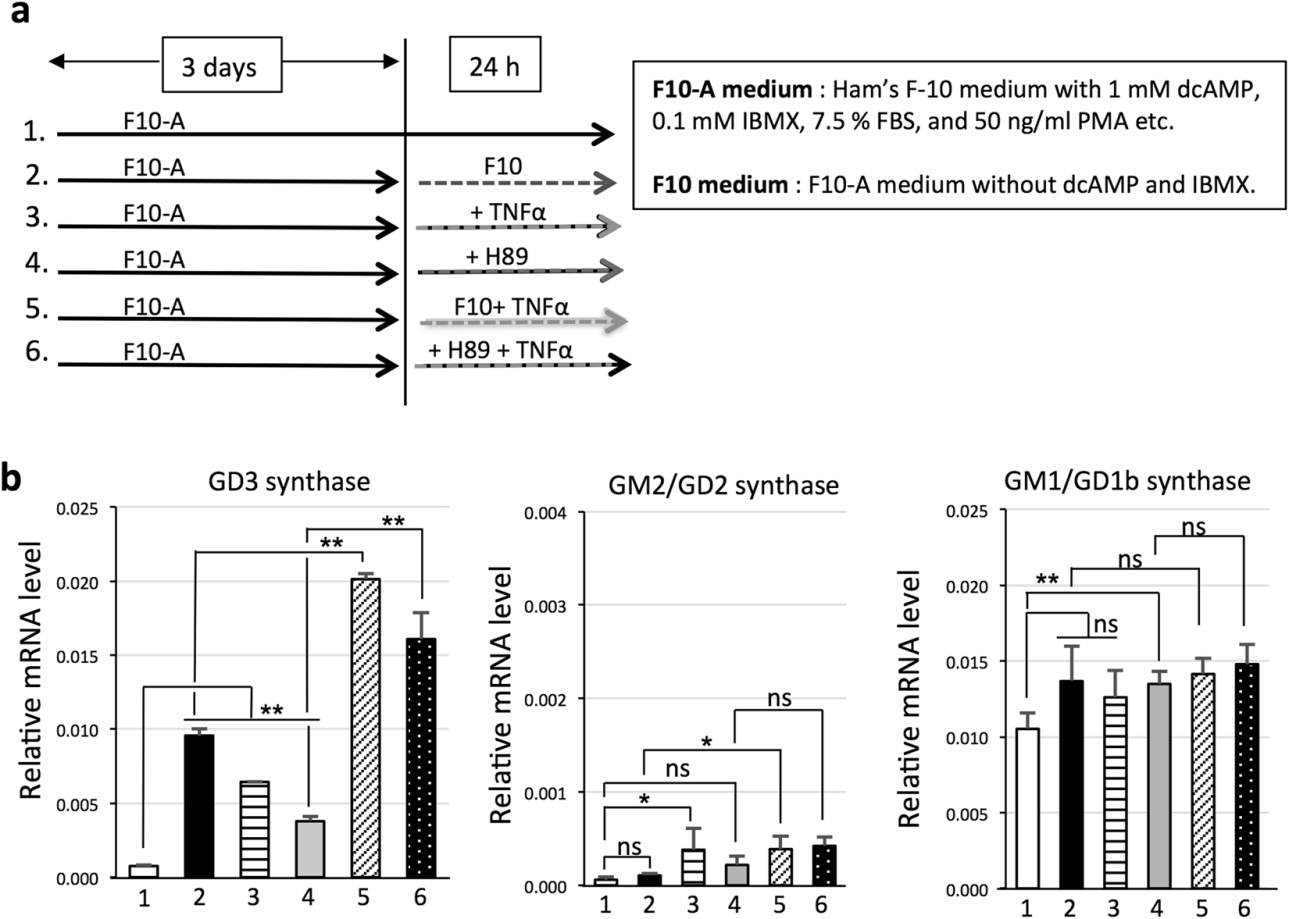
Combined treatment of melanocytes with TNFα and H89 or TNFα and removal of dcAMP further enhances expression of the GD3 synthase gene. (**a**) Melanocytes (LP) were cultured according to protocols 1~6. Melanocytes were cultured in F10-A medium for 3 days. Then, 1. melanocytes were cultured in F10-A medium for 24 h, 2. cultured in F10-A medium without dcAMP and IBMX (F10 medium) for 24 h, 3. treated with TNFα (10 ng/mL) for 24 h, 4. treated with H89 (10 μM) for 24 h, 5. Melanocytes were cultured in F10 medium with TNFα (10 ng/mL) for 24 h, and 6. cultured with TNFα (10 ng/mL) and H89 (10 μM) for 24 h. (**b**) Melanocytes were cultured according to protocols 1~6 as in (**a**). mRNA expression levels of GD3 synthase, GM2/GD2 synthase, and GM1/GD1b synthase were analyzed by qRT-PCR. mRNA expression levels of these glycosyltransferases were normalized by those of human GAPDH gene. Data represent means ± s.d. (n = 4). Statistical analysis was performed by the two-tailed Student’s t-test (**P < 0.01).

Melanocytes were cultured in F10-A medium for 3 days, and then we prepared melanocytes cultured under various conditions. Melanocytes were cultured by protocols 1~6 (p1~p6) (Fig. 4a). GD3 synthase gene expression was increased by all of the following treatments, i.e., removal of dcAMP (1 mM) and IBMX (0.1 mM) from the culture medium (p2), addition of TNFα (10 ng/mL) (p3), or addition of H89 (10 μM) for 24 h (p4) when compared with the untreated sample (p1). Further, the combination of TNFα and removal of dcAMP and IBMX (p5) enhanced GD3 synthase gene expression more than treatments with either one of them alone. Similarly, the combination of TNFα and H89 (p6) more markedly enhanced GD3 synthase gene expression than treatment with a single reagent (Fig. 4b). mRNA expression levels of GM1/GD1b synthase showed almost no change, while those of GM2/GD2 synthase tended to slightly increase by treatment with any of the protocols (p2~p4) in Fig. 4a.

### Treatment with an IκB kinase (IKK) inhibitor, wedelolactone (WDL), reduces expression level of the GD3 synthase gene induced by TNFα in melanocytes

**Figure 5:**
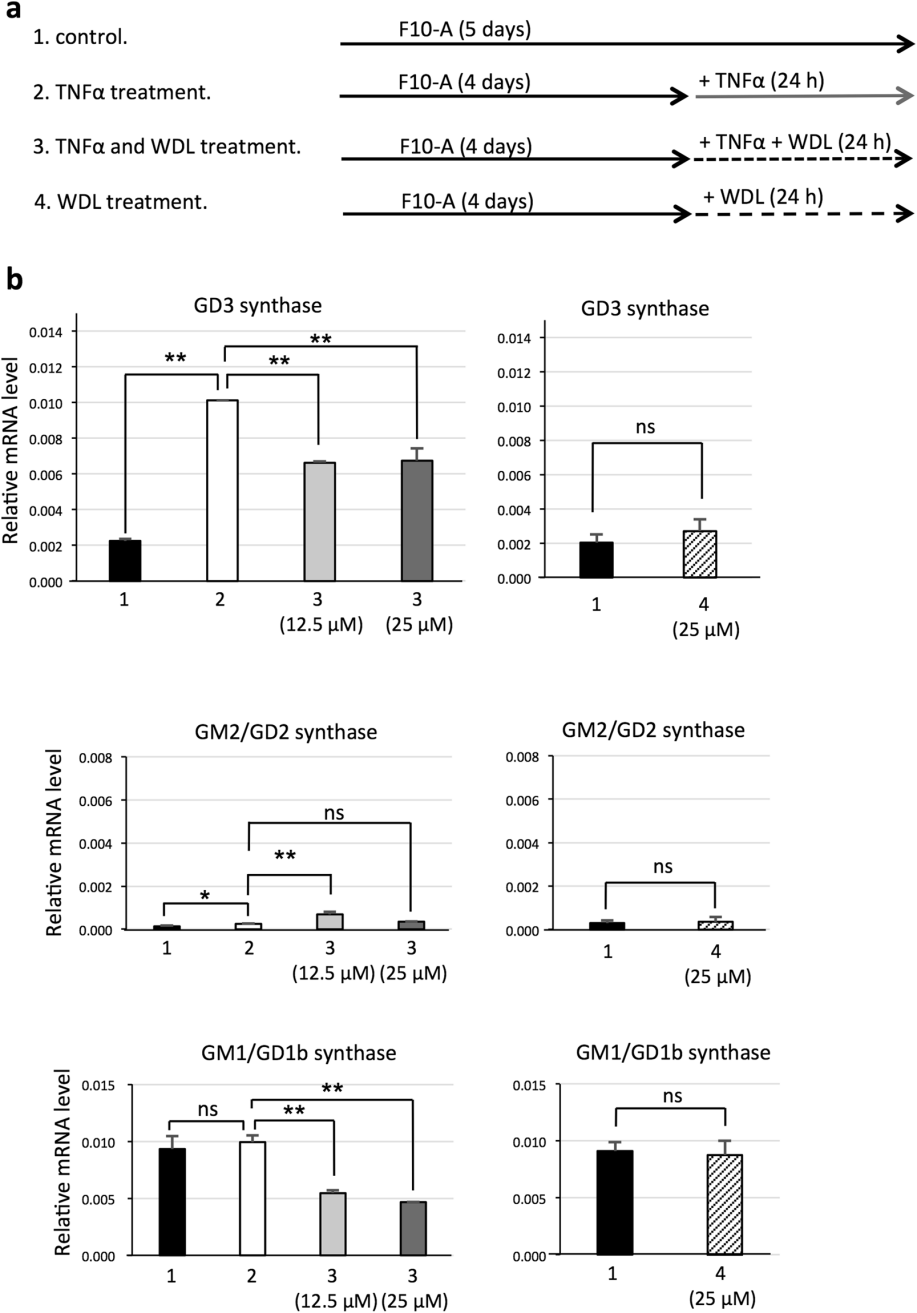
Inhibition of IKK results in suppression of GD3 synthase gene expression induced by TNFα in melanocytes. (**a**) Melanocytes (LP) were cultured according to protocols 1~4. Melanocytes were cultured in F10-A medium for 4 days. Then, 1. melanocytes were cultured in F10-A medium for 24 h, 2. cultured with TNFα (10 ng/mL) for 24 h, 3. cultured with TNFα (10 ng/mL) and an IKK inhibitor, WDL (12.5 μM or 25 μM), for 24 h, and 4. cultured with WDL (25 μM). (**b**) After treatment of melanocytes with or without TNFα and WDL as in (**a**), mRNA expression levels of GD3 synthase, GM2/GD2 synthase, and GM1/GD1b synthase were analyzed by qRT-PCR. mRNA expression levels of these glycosyltransferases were normalized by those of human GAPDH gene. Data represent means ± s.d. (n = 4). Statistical analysis was performed by the two-tailed Student’s t-test (*P < 0.05; **P < 0.01). ns = not significant.

Melanocytes were cultured in the medium as indicated in Fig. 5a and an IKK inhibitor, WDL was used to confirm that IKKα and β (IKKα/β) which are downstream molecules of TNFα receptor were involved in the GD3 synthase gene expression. While the GD3 synthase gene expression level was increased by TNFα treatment, as described above, it was suppressed by concomitant treatment with TNFα (10 ng/mL) and WDL (12.5 or 25 μM) (Fig. 5b). GM2/GD2 synthase gene expression levels were moderately increased at low levels when treated with TNFα or TNFα and WDL. Surprisingly, GM1/GD1b synthase gene expression levels were significantly reduced by WDL treatment. When melanocytes were treated with WDL alone, the gene expression levels of GD3 synthase, GM2/GD2 synthase, and GM1/GD1b synthase were almost same as thouse without WDL (Fig. 5b). WDL was dissolved in DMSO and added to the culture medium (12.5 or 25 μM as a final concentration). The solvent, DMSO showed no effect on relative mRNA levels (Supplementary Fig. S3).

We confirmed that GD3 synthase gene expression in other melanocytes, HEMn-MP, was also induced by TNFα treatment and suppressed by concomitant treatment with TNFα and WDL (Supplementary Fig. S6).

### Addition of TNFα, H89, and/or dcAMP dose not affect expression levels of glycosyltransferases in melanomas

**Figure 6:**
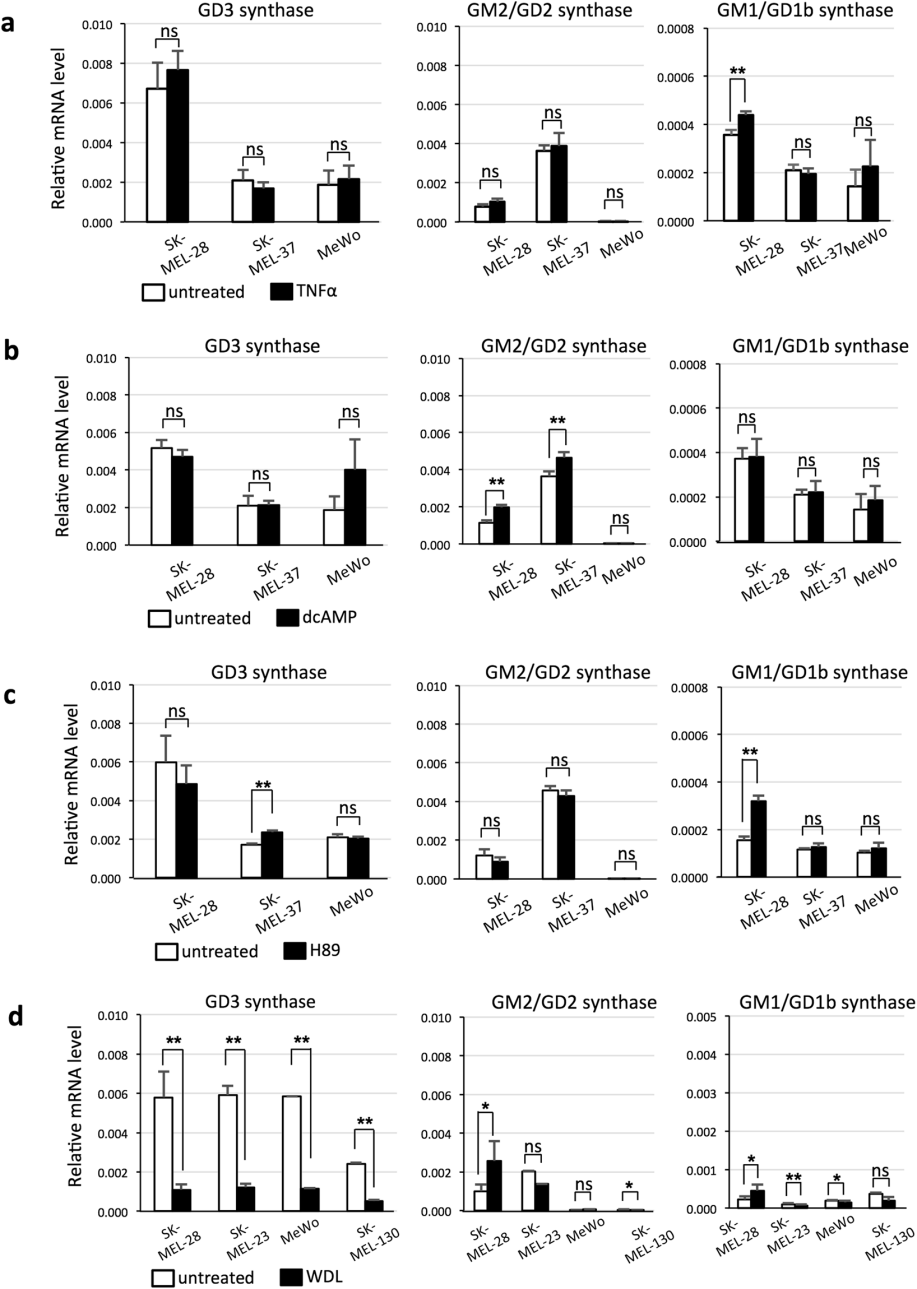
dcAMP, TNFα, and H89 do not affect expression levels of glycosyltransferases, while WDL suppresses GD3 synthase gene expression in melanomas. Melanoma cell lines, SK-MEL-28, SK-MEL-37, and MeWo were treated with or without TNFα (10 ng/mL) (**a**), dcAMP (1 mM) (**b**), and H89 (10 μM) (**c**) for 24 h. Then, mRNA expression levels of GD3 synthase, GM2/GD2 synthase, and GM1/GD1b synthase were analyzed by qRT-PCR. mRNA expression levels of these glycosyltransferases were normalized by those of human GAPDH gene. Data represent means ± s.d. (n = 4). (**d**) Treatment of melanoma cell lines (SK-MEL-28, SK-MEL-23, MeWo, and SK-MEL-130) with WDL reduced expression levels of the GD3 synthase gene. After treatment with or without WDL (20 μM) for 24 h, mRNA expressions levels of GD3 synthase, GM2/GD2 synthase, and GM1/GD1b synthase were analyzed by qRT-PCR. mRNA expression levels of these glycosyltransferases were normalized by those of human GAPDH gene. Data represent means ± s.d. (n = 3–4). Statistical analysis was performed by the two-tailed Student’s t-test (*P < 0.05; **P < 0.01). ns = not significant.

In melanocytes, the addition of TNFα or H89 increased the GD3 synthase gene expression level, and addition of dcAMP suppressed it. Then, we examined the effects of TNFα (10 ng/mL), dcAMP (1 mM), and H89 (10 μM) on melanoma cell lines. Consequently, none of them affected GD3 synthase gene expression levels in melanoma cells (Fig. 6a–c). Similarly, TNFα, dcAMP, and H89 treatments did not consistently affect mRNA expression levels of GM2/GD2 synthase or GM1/GD1b synthase genes in melanoma cells.

### Treatment with an IKK inhibitor, WDL, significantly suppresses GD3 synthase gene expression levels in melanomas

On the other hand, WDL (20 μM) treatment significantly suppressed the GD3 synthase gene expression level in melanoma cell lines (Fig. 6d). As for expression levels of GM2/GD2 synthase and GM1/GD1b synthase genes, no consistent changes were observed, similarly to treatment with other reagents.

### WDL treatment results in reduced IKK phosphorylation levels in melanocytes and melanoma cells

**Figure 7:**
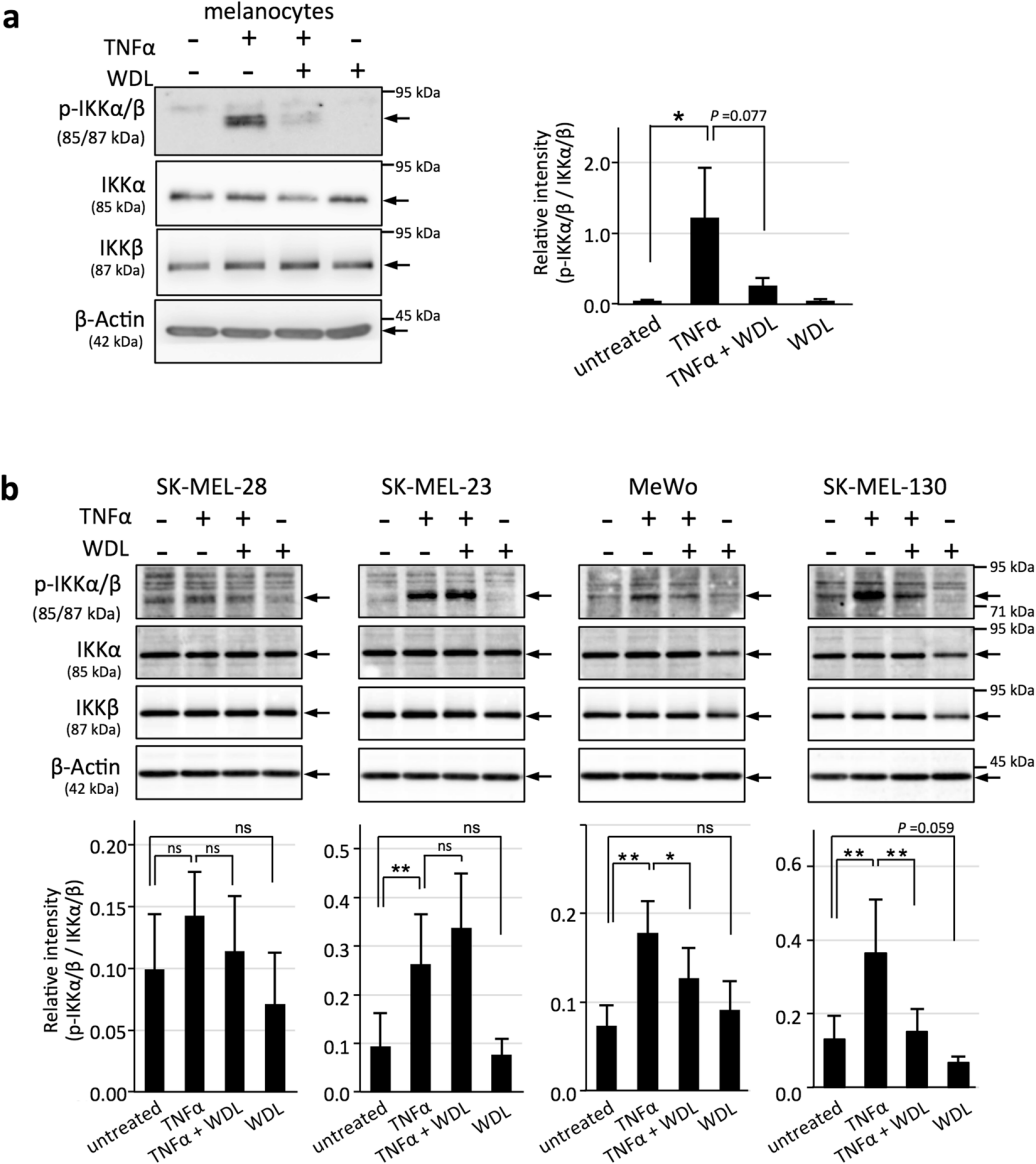
Inhibition of phosphorylation of IKK by WDL in melanocytes and melanomas. (**a**) Melanocytes (LP) were treated with TNFα (10 ng/mL), TNFα and WDL (20 μM), or WDL alone for 24 h. Data represent means ± s.d. (n = 3). (**b**) SK-MEL-28, SK-MEL-23, MeWo, and SK-MEL-130 were treated with TNFα (10 ng/mL), TNFα and WDL (20 μM), or WDL alone for 24 h. Phosphorylation levels of IKKs (p-IKKα/β) were examined by western blotting with anti-phospho-IKKα/β (Ser176/180) antibody. Band intensities of p-IKKα/β were measured and presented after correction by those of IKKα/β. Four kinds of samples from melanocytes and individual melanoma cell lines (TNFα (−)/WDL (−), TNFα (+)/WDL (−), TNFα (+)/WDL (+), TNFα (−)/WDL (+)) were applied on the same gel and immunoblotted. Data represent means ± s.d. (n = 5–7). Statistical analysis was performed by the two-tailed Student’s t-test (*P < 0.05; **P < 0.01). ns = not significant.

In melanocytes, IKKs were scarcely phosphorylated under the standard culture conditions. TNFα treatment markedly induced or enhanced phosphorylation of IKKα/β. When TNFα (10 ng/mL) was added together with WDL (20 μM), the phosphorylation levels of IKKα/β decreased (Fig. 7a). WDL alone did not affect the level of IKKα/β phosphorylation. In melanoma cells, low levels of phosphorylation of IKKα/β were detected before TNFα treatment, and TNFα (10 ng/mL) treatment enhanced phosphorylation of IKKα/β in melanoma cells except SK-MEL-28. In SK-MEL-28, the phosphorylation level of IKKα/β without TNFα treatment was high and elevation of the phosphorylation level with TNFα treatment was not clear compared with other melanoma cell lines. WDL (20 μM) treatment markedly reduced increased phosphorylation levels of IKKα/β with the addition of TNFα in MeWo and SK-MEL-130, but did not necessarily reduce phosphorylation levels of IKKα/β in SK-MEL-28 or SK-MEL-23. WDL treatment without TNFα tended to reduce phosphorylation levels of IKKα/β compared with those with no treatment (Fig. 7b).

**Figure 8:**
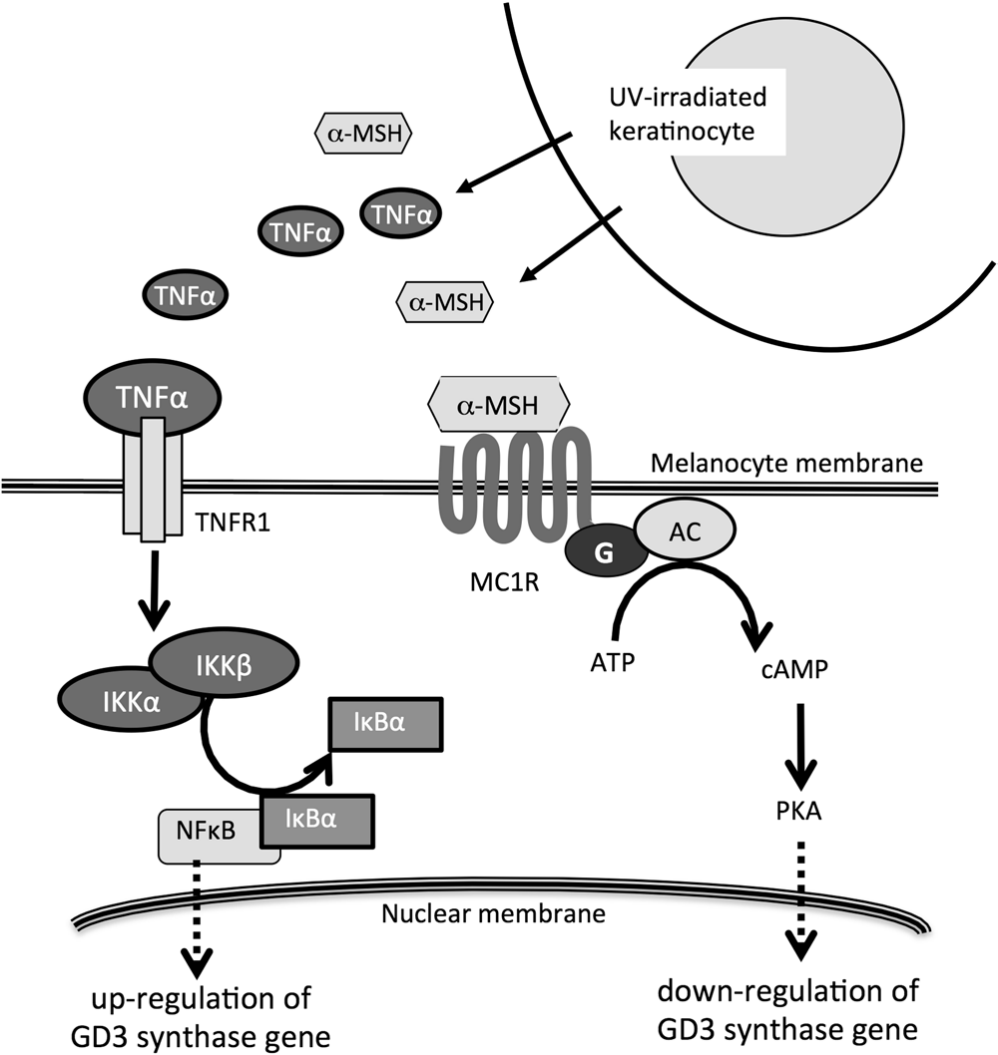
TNFα and cAMP-PKA signal pathways exert opposite effects to regulate GD3 synthase gene expression in human melanocytes. The TNFα signal pathway upregulated expression of the GD3 synthase gene, while cAMP-PKA signaling suppressed its expression.

In Fig. 8, a summary of the results of this study is shown. TNFα and α-MSH stimulate melanocytes, inducing the activation of NF-κB and PKA-mediated signals, respectively. These signaling pathways regulate GD3 synthase gene expression in opposite directions, with either one being dominant depending on the situations.

## Discussion

Malignant melanomas are considered to be derived from melanocytes, and to be induced by various intrinsic and extrinsic factors such as UV-irradiation^22^. As for expression patterns of gangliosides, melanocytes and melanoma cells showed distinct patterns, i.e. melanomas universally express ganglioside GD3 as a melanoma-associated glycolipid antigen^3,23^, and sometimes express GM2 and GD2, while melanocytes mainly express GM3 and scarcely express GD3^9^. Sumantran *et al*. re-analyzed an existing microarray dataset (GDS-1375) to identify differentially expressed genes, in normal skin, benign nevi, and cutaneous melanomas. Out of 18 genes that can promote lipogenesis in melanomas, 4 genes for the synthesis of gangliosides were upregulated in melanomas^24^. The ST8 alpha-N-acetyl-neuraminide alpha-2, 8-sialyltransferase 1 (*ST8SIA 1*) gene was the most upregulated in melanoma cell lines in NCI-60 Cell Miner database. Our results in this study that the *ST8SIA 1* gene was clearly upregulated in melanoma cell lines is consistent with the above-mentioned results^24^.

Regulatory mechanisms for gene expression of ganglioside synthases in melanomas have been analyzed by some studies including our group^25,26^. However, those in melanocytes have never been analyzed. In particular, no information is available on regulation of the GD3 synthase gene in melanocytes, while its regulation by NF-κB in melanomas was reported^27^.

In this study, we analyzed expressions of ganglioside synthase genes using multiple melanocyte lines and melanoma cell lines. Consequently, the GD3 synthase gene (*ST8SIA1*) was highly expressed only in the melanoma cell lines examined, and GM2/GD2 synthase (*B4GALNT1*) was also expressed only in melanoma cells (Fig. 1b). As we previously reported^10^, GD3 synthase gene expression was markedly induced by TNFα in two melanocyte lines (LP and MP), while expression of GM2/GD2 synthase gene moderately increased and that of GM1/GD1b synthase gene was not affected (Fig. 2a and Supplementary Fig. S6). These results indicated that GD3 synthase is specifically induced by inflammatory cytokines in melanocytes, as previously reported^10^.

Then, we examined the effects of removing dcAMP and a phosphodiesterase inhibitor, IBMX, which are considered to be an activator of protein kinase A (PKA) and inhibitor of cAMP degradation, respectively, on the expression levels of glycosyltransferase genes in melanocytes. Consequently, the elimination of dcAMP and IBMX resulted in induction of the GD3 synthase gene, but not GM2/GD2 synthase or GM1/GD1b synthase genes (Fig. 3b). Here, opposite actions of TNFα and PKA in GD3 synthase gene regulation were revealed. This result was also confirmed by the addition of α-MSH during the removal of dcAMP and IBMX, showing suppression of the GD3 synthase mRNA level elevated by elimination of dcAMP and IBMX. Furthermore, treatment of melanocytes under culture in F10-A (containing dcAMP and IBMX) by a PKA inhibitor, H89, resulted in a definite increase of GD3 synthase gene expression (Fig. 3f and Supplementary Fig. S5). All these results suggest that cAMP-mediated signals suppress GD3 synthase gene expression via PKA.

Melanocytes have been considered to be a multi-potent player with roles beyond simple pigment production in skin tissues, i.e., a member of the skin immune system and a key player in an epidermal stress-response system^28^. When keratinocytes undergo UV irradiation, POMC (proopiomelanocortin) is produced, and POMC is cleaved into several peptides including α-MSH by PC1/2 (protein convertases 1/2) in keratinocytes. Then, α-MSH is released from keratinocytes toward melanocytes^29^. Regulation of melanocytes is mainly performed by α-MSH via the melanocortin-1 receptor (MC1R)^30^, leading to the activation of adenylate cyclase and generation of cAMP^23,31^. Among various functions of α-MSH, following their unique aspects may be important: anti-inflammatory function^32^, suppression of oxidative damage^33^, protection against and repair of DNA damage^34–36^, melanocyte differentiation^23^, and photoprotection via pigmentation^37^, have been extensively investigated^38^.

UVB-irradiated keratinocytes secrete both inflammatory cytokines and α-MSH, leading to different phenotypes of melanocytes (Fig. 8). These two factors trigger distinct intracellular signaling pathways, resulting in different directions of ganglioside synthetic pathways in melanocytes, as shown here. The significance of these opposite signals in melanocyte physiology is a key question, and how these systems are orchestrated in melanocytes is an intriguing research issue to promote understanding of melanomagenesis.

Generally, the significance of GD3 and/or GD3 synthase expression has been considered to be either a sign of cell activation^6,39^, prediction of transformation^40^, an indicator of potential stem cells in the nervous system^41,42^, an urgent responder to critical changes of microenvironments^10^ or apoptosis^43^. Recently, a report indicated that GD3-enriched extracellular vesicles (EVs) derived from GD3-expressing melanocytes stimulated the migration of GD3-negative cells^20^. At least in the melanocyte-melanoma context, the present results may substantiate roles of GD3/GD3 synthase in the transformation of melanocytes and development to melanomas by opposing effects of α-MSH-MC1R-cAMP-mediated signals^23,38^. In turn, the α-MSH-MC1R axis plays critical roles in the regulation of inflammation^28,32^, pigmentation^44,45^, cell cycle^46^, DNA damage response^23^, and apoptosis^38^. Many mutants of the MC1R gene (~200 mutants in the coding region) and the results from MC1R-knockout mice suggest that MC1R is a melanoma susceptibility gene^47,48^. Actually, α-MSH and MC1R are being considered to be melanocyte-protecting factors from malignant transformation^38^. Recently, Chen *et al*. reported that some MC1R variants have less-active MC1R signaling^49^. TNFα signaling in the melanocytes that have such a MC1R variants might become dominant, although keratinocytes release both α-MSH and TNFα by UVB radiation^10,29^, and both of them stimulate adjacent melanocytes. Consequently, in the absence of cAMP signaling, expression levels of GD3 synthase gene and GD3 might markedly increase as shown in Fig. 4. Thus, increased expression of GD3 synthase gene and/or GD3 in melanocytes may become a useful indicator of precancerous state and/or chronic inflammation. Recently, enhancement of the migration of melanocytes due to increased GD3 expression was reported by Otake *et al*.^20^.

Although it is well-known that keratinocytes and/or melanocytes generate and excrete inflammatory cytokines^10,32,50^ and also α-MSH, how these two signalings, pre-inflammatory and anti-inflammatory/oxidation/DNA damage signals, are differentially regulated remains to be clarified. Namely, how either signal becomes dominant in what kind of situations may be critical. Levels of stresses such as UV-irradiation, and the frequency or intervals and/or qualities of those stresses should be carefully investigated to clarify cross-talk between signals promoting or suppressing GD3 synthase expression, leading to further understanding of the roles of GD3 synthase in melanomagenesis^51^.

In contrast to GD3 and GD3 synthase, GM3 and GM1 or their synthase genes have been considered to be contrary players involved in regulation of the cell state, i.e., regulatory and suppressive functions to stabilize cell conditions^18^. Whether these monosialyl gangliosides significantly exert their roles in melanocytes to stabilize genome states and cell signaling remains to be investigated in the near future.

There should be definite differences in the regulatory mechanisms for the expression of gangliosides and their synthetic enzymes between melanocytes and melanoma cells. It may be particularly important to examine the difference in their sensitivity to α-MSH and TNFα, and involvement of NF-κB in the regulation of ganglioside synthases. The application of those differences in melanoma therapeutics may be a promising strategy, and preliminary trials using a mouse xenograft model are being prepared in our laboratory.

## Methods

### Cell culture

HEMn-LP, a lightly pigmented normal human melanocyte line (LP) and HEMn-MP, a moderately pigmented normal human melanocyte line (MP), were purchased from Thermo Fisher Scientific (Yokohama, Japan), and cultured in Medium 254 supplemented with Human Melanocyte Growth Supplement^TM^ (HMGS) (Thermo Fisher Scientific, Yokohama, Japan). When 70∼80% confluency was reached, the culture medium was changed from Medium 254 with HMGS to Ham’s F-10 medium supplemented with 7.5% fetal bovine serum (FBS), 1% penicillin–streptomycin, 1 mM N^6^, 2′-O-dibutyryladenosine 3′, 5′-cyclic monophosphate sodium salt (dcAMP), 0.1 mM 3-isobutyl-1-methylxanthine (IBMX), 1 μM Na_3_VO_4_, and phorbol 12-myristate 13-acetate (PMA) (50 ng/ml) (F10-A medium)^52^. Ham’s F-10 medium and penicillin–streptomycin were purchased from Life Technologies, FBS from Equitech-Bio (Kerrville, TX, USA), and all others from Sigma–Aldrich (St. Louis, MO, USA). After 4-to-7-day incubation, cells were used for experiments. All human melanoma cell lines were provided by Dr. L. J. Old (Memorial Sloan-Kettering Cancer Center, New York, USA) and cultured in Dulbecco’s modified Eagle’s essential medium (D-MEM) supplemented with 7.5% FBS.

### Reagents

Recombinant human TNFα, [Nle^4^, D-Phe^7^]-alpha-melanocyte-sitmulating hormone trifluoroacetate salt (α-MSH) was purchased from Sigma–Aldrich (St. Louis, MO). InSolution^TM^ H-89, dihydrochloride, and an IKK inhibitor, Wedelolactone^TM^ were purchased from MERCK MILLIPORE (Burlington, MA, USA). The optimal concentrations of these reagents were determined by titration and used for experiments (Supplementary Fig. S7).

### Real-time reverse transcription-polymerase chain reaction (RT-PCR)

Total RNA was prepared using RNeasy Plus Mini™ Kit (QIAGEN, Hilden, Germany) from all melanocytes and melanoma cell lines. cDNA synthesis from total RNA was performed using iScript™ Advanced cDNA Synthesis Kit (Bio-Rad Laboratories, Hercules, CA, USA). Quantitative RT-PCR (qRT-PCR) analysis was performed using iTaq™ Universal SYBR Green Supermix™ (Bio-Rad). Primers used in this study were designed with Probe Finder™ software (Roche Diagnostics, Basel, Switzerland) and primer sequences for the genes were as follows: *ST8SIA1*: forward (5′-GGAAATGGTGGGATTCTGAAG-3′), reverse (5′-TGACAAAGGAGGGAGATTGC-3′), *B4GALNT1*: forward (5′-CCAACTCAACAGGCAACTACAA-3′), reverse (5′-ATGTCCCTCGGTGGAGAAC-3′); *B3GALT4*: forward (5′-TGCTGCAGTTGTTCTCTCAAG-3′), reverse (5′-AAGTTTATTGAGGAGCTTGACACC-3′); *hACTB*: forward (5′-CCAACCGCGAGAAGATGA-3′), reverse (5′-CCAGAGGCGTACAGGGATAG-3′); *hGAPDH*: forward (5′-GTCAGTGGTGGACCTGACCT-3′), reverse (5′-TGCTGTAGCCAAATTCGTTG-3′).

### Immunofluorescence staining

Melanocytes were fixed in 4% paraformaldehyde for 30 min at room temperature, and blocked with 2% BSA in PBS overnight at 4 °C. Then, GD3 was stained with anti-GD3 mAb (R24) and Alexa 594-conjugated donkey anti-mouse IgG (Thermo Fisher Scientific, Waltham, MA, USA). Cells were imaged using a confocal microscope (FLUOVIEW Fv10^TM^, OLYMPUS, Tokyo, Japan).

### Western immunoblotting

Cells were lysed with cell lysis buffer (20 mM Tris-HCl, 150 mM NaCl, 1 mM Na_2_EDTA, 1 mM EGTA, 1% Triton X-100, 2.5 mM sodium pyrophosphate, 1 mM β-glycerophosphate, 1 mM Na_3_VO_4_, 1 mM leupeptin) (Cell Signaling Technology, Danvers, MA, USA) added with 1 mM PMSF or RIPA lysis buffer (50 mM Tris-HCl, 1% NP40, 0.1% SDS, 0.5% sodium deoxycholate, 150 mM NaCl, 1 mM EDTA, 1 mM Na_3_VO_4_, and 1 mM NaF) added with 1 mM PMSF and Protease Inhibitor Cocktail Set I (Merk Millipore, Burlington, MA, USA). Insoluble materials were removed by centrifugation at 10,000 × *g* for 10 min at 4°C. Cell lysates were separated by SDS-PAGE using 10% gels. The separated proteins were transferred onto an Immunobilon-P^TM^ membrane (Millipore, Billerica, MA, USA). Blots were blocked with 5% skim milk in Tris-buffered saline containing 0.05% Tween 20 or 3% BSA in PBS containing 0.05% Tween 20 for 1 h at room temperature, and incubated with anti-IKKα, anti-IKKβ antibody, or anti-Phospho-IKKα/β (Ser176/180) antibody (Cell Signaling Technology) as primary antibodies. Antibodies used for detection of glycosyltransferases were as follows, GD3 Synthase (B-11) (a mouse monoclonal antibody) (Santa Cruz Biotechnology, Inc., Dallas, TX, USA); GM2/GD2 Synthase (C-5) (a mouse monoclonal antibody) (Santa Cruz Biotechnology, Inc.); B3GALT4 (GM1/GD1b synthase) Polyclonal Antibody (Proteintech Group, Inc., Rosemont, IL, USA). Monoclonal antibodies used for detection of CREB and phospho-CREB were as follows, CREB (48H2) Rabbit mAb (Cell Signaling Technology); Phospho-CREB (Ser133) (87G3) Rabbit mAb (Cell Signaling Technology). After being washed, the blots were incubated with anti-mouse or rabbit IgG conjugated with horseradish peroxidase. After washing, bound conjugates on the membrane were visualized with an Enhanced Chemiluminescence^TM^ detection system (PerkinElmer Life Sciences, Waltham, MA, USA) or ImmunoStar® LD (Wako Pure Chemical, Osaka, Japan). Chemiluminescence was detected and analyzed by Image Quant^TM^ LAS 4000 (GE Healthcare Bio-Sciences AB, Uppsala, Sweden) or Amersham Imager 680^TM^ (GE Healthcare UK Ltd., Buckinghamshire, UK).

### Statistical analysis

Data are presented as means ± standard deviation (s.d.) of individual experiments. Results were initially analyzed for homogeneity of variance using Bartlett’s, Hartley’s, and Levene’s tests. The Shapiro-Wilk test was used to verify that the data showed a normal distribution. Significance was calculated using the two-tailed Student’s t-test. All significances levels were set as *P < 0.05; **P < 0.01.

## Supplementary information

Supplementary

## Data Availability

All data are available.

## References

[1] Merimsky O, Shoenfeld Y, Chaitchik S, Yecheskel G, Fishman P (1994). Antigens and antibodies in malignant melanoma. Tumour Biol.

[2] Furukawa K., Lloyd K. O. (1990). Gangliosides in Melanoma. Human Melanoma.

[3] Portoukalian J, Zwingelstein G, Doré JF (1979). Lipid composition of human malignant melanoma tumors at various levels of malignant growth. Eur J Biochem.

[4] Carubia JM, Yu RK, Macala LJ, Kirkwood JM, Varga JM (1984). Gangliosides of normal and neoplastic human melanocytes. Biochem Biophys Res Commun.

[5] Dippold WG (1980). Cell surface antigens of human malignant melanoma: definition of six antigenic systems with mouse monoclonal antibodies. Proc Natl Acad Sci USA.

[6] Yamashiro S (1995). Expression of alpha 2,8-sialyltransferase (GD3 synthase) gene in human cancer cell lines: high level expression in melanomas and up-regulation in activated T lymphocytes. Glycoconj J.

[7] Watanabe T (1982). Human melanoma antigen AH is an autoantigenic ganglioside related to GD2. J. Exp Med.

[8] Tai T, Cahan LD, Paulson JC, Saxton RE, Irie RF (1984). Human monoclonal antibody against ganglioside GD2: use in development of enzyme-linked immunosorbent assay for the monitoring of anti-GD2 in cancer patients. J. Natl Cancer Inst.

[9] Thampoe IJ, Furukawa K, Vellvé E, Lloyd KO (1989). Sialyltransferase levels and ganglioside expression in melanoma and other cultured human cancer cells. Cancer Res.

[10] Miyata M (2014). UVB-irradiated keratinocytes induce melanoma-associated ganglioside GD3 synthase gene in melanocytes via secretion of tumor necrosis factor α and interleukin 6. Biochem Biophys Res Commun.

[11] Furukawa K (2017). Glycolipids: Essential regulator of neuro-inflammation, metabolism and gliomagenesis. Biochim Biophys Acta.

[12] Hamamura K (2005). Ganglioside GD3 promotes cell growth and invasion through p130Cas and paxillin in malignant melanoma cells. Proc Natl Acad Sci USA.

[13] Ohkawa Y (2015). Ganglioside GD3 Enhances Invasiveness of Gliomas by Forming a Complex with Platelet-derived Growth Factor Receptor α and Yes Kinase. J. Biol Chem.

[14] Iwasawa T (2018). Enhancement of malignant properties of human glioma cells by ganglioside GD3/GD2. Int J Oncol.

[15] Cazet A (2012). The ganglioside G(D2) induces the constitutive activation of c-Met in MDA-MB-231 breast cancer cells expressing the G(D3) synthase. Glycobiology.

[16] Yoshida S (2001). Ganglioside G(D2) in small cell lung cancer cell lines: enhancement of cell proliferation and mediation of apoptosis. Cancer Res.

[17] Shibuya H (2012). Enhancement of malignant properties of human osteosarcoma cells with disialyl gangliosides GD2/GD3. Cancer Sci.

[18] Furukawa K (2012). Fine tuning of cell signals by glycosylation. J. Biochem.

[19] Ohmi Yuhsuke, Kambe Mariko, Ohkawa Yuki, Hamamura Kazunori, Tajima Orie, Takeuchi Rika, Furukawa Koichi, Furukawa Keiko (2018). Differential roles of gangliosides in malignant properties of melanomas. PLOS ONE.

[20] Otake AH (2019). GD3 ganglioside-enriched extracellular vesicles stimulate melanocyte migration. Biochim Biophys Acta Mol Cell Biol Lipids..

[21] Rodríguez CI, Setaluri V (2014). Cyclic AMP (cAMP) signaling in melanocytes and melanoma. Arch Biochem Biophys..

[22] Lo JA, Fisher DE (2014). The melanoma revolution: from UV carcinogensis to a new era in therapeutics. Science.

[23] Pukel CS (1982). GD3, a prominent ganglioside of human melanoma. Detection and characterisation by mouse monoclonal antibody. J. Exp Med.

[24] Sumantran VN, Mishra P, Sudhakar N (2015). Microarray analysis of differentially expressed genes regulating lipid metabolism during melanoma progression. Indian J. Biochem. Biophys..

[25] Furukawa K, Soejima H, Niikawa N, Shiku H (1996). Genomic organization and chromosomal assignment of the human beta1, 4-N-acetylgalactosaminyltransferase gene. Identification of multiple transcription units. J. Biol Chem.

[26] Furukawa K, Horie M, Okutomi K, Sugano S, Furukawa K (2003). Isolation and functional analysis of the melanoma specific promoter region of human GD3 synthase gene. Biochim Biophys Acta.

[27] Kang NY (2006). Transcriptional regulation of the human GD3 synthase gene expression in Fas-induced Jurkat T cells: a critical role of transcription factor NF-kappaB in regulated expression. Glycobiology.

[28] Plonka PM (2009). What are melanocytes really doing all day long…?. Exp Dermatol.

[29] Chakraborty AK (1996). Production and release of proopiomelanocortin (POMC) derived peptides by human melanocytes and keratinocytes in culture: regulation by ultraviolet B. Biochim Biophys Acta.

[30] Lin JY, Fisher DE (2007). Melanocyte biology and skin pigmentation. Nature.

[31] Rosenkranz AA, Slastnikova TA, Durymanov MO, Sobolev AS (2013). Malignant melanoma and melanocortin 1 receptor. Biochemistry (Mosc).

[32] Haycock JW (1999). Alpha-melanocyte-stimulating hormone inhibits NF-kappaB activation in human melanocytes and melanoma cells. J. Invest Dermatol.

[33] Kadekaro AL (2005). Alpha-Melanocortin and endothelin-1 activate antiapoptotic pathways and reduce DNA damage in human melanocytes. Cancer Res.

[34] D’Orazio JA (2006). Topical drug rescue strategy and skin protection based on the role of Mc1r in UV-induced tanning. Nature.

[35] Smith AG (2008). Melanocortin-1 receptor signaling markedly induces the expression of the NR4A nuclear receptor subgroup in melanocytic cells. J. Biol Chem.

[36] Kadekaro AL (2010). Melanocortin 1 receptor genotype: an important determinant of the damage response of melanocytes to ultraviolet radiation. FASEB J.

[37] Meyskens FL, Farmer P, Fruehauf JP (2001). Redox regulation in human melanocytes and melanoma. Pigment Cell Res.

[38] García-Borrón JC, Abdel-Malek Z, Jiménez-Cervantes C (2014). MC1R, the cAMP pathway, and the response to solar UV: extending the horizon beyond pigmentation. Pigment Cell Melanoma Res.

[39] Amat JA, Ishiguro H, Nakamura K, Norton WT (1996). Phenotypic diversity and kinetics of proliferating microglia and astrocytes following cortical stab wounds. Glia.

[40] Albino AP (1986). Class II histocompatibility antigen expression in human melanocytes transformed by Harvey murine sarcoma virus (Ha-MSV) and Kirsten MSV retroviruses. J. Exp Med.

[41] Yeh SC (2016). Glycolipid GD3 and GD3 synthase are key drivers for glioblastoma stem cells and tumorigenicity. Proc Natl Acad Sci USA.

[42] Nakatani Y, Yanagisawa M, Suzuki Y, Yu RK (2010). Characterization of GD3 ganglioside as a novel biomarker of mouse neural stem cells. Glycobiology.

[43] De MR (1997). Requirement for GD3 ganglioside in CD95- and ceramide-induced apoptosis. Science.

[44] Abdel-Malek Z (1995). Mitogenic and melanogenic stimulation of normal human melanocytes by melanotropic peptides. Proc Natl Acad Sci USA.

[45] Dumaz N (2006). In melanoma, RAS mutations are accompanied by switching signaling from BRAF to CRAF and disrupted cyclic AMP signaling. Cancer Res.

[46] Lyons. J, Bastian BC, McCormick F (2013). MC1R and cAMP signaling inhibit cdc25B activity and delay cell cycle progression in melanoma cells. Proc Natl Acad Sci USA.

[47] Kennedy C (2001). Melanocortin 1 receptor (MC1R) gene variants are associated with an increased risk for cutaneous melanoma which is largely independent of skin type and hair color. J. Invest Dermatol.

[48] Ichii-Jones F (1998). Susceptibility to melanoma: influence of skin type and polymorphism in the melanocyte stimulating hormone receptor gene. J. Invest Dermatol.

[49] Chen S (2017). Palmitoylation-dependent activation of MC1R prevents melanomagenesis. Nature..

[50] Slominski A (2005). CRH stimulation of corticosteroids production in melanocytes is mediated by ACTH. Am J Physiol Endocrinol Metab.

[51] Furukawa K (2017). Inflammatory reactions in microenvironments, leading to melanomagenesis. J. Clin. Cell. Immunol..

[52] McGill GG, Haq R, Nishimura EK, Fisher DE (2006). c-Met expression is regulated by Mitf in the melanocyte lineage. J. Biol Chem..

