# Supplementary material for: TNFα-signal and cAMP-mediated signals oppositely regulate melanoma- associated ganglioside GD3 synthase gene in human melanocytes
Source: Sci Rep. 2019 Oct 14;9:14740. doi: 10.1038/s41598-019-51333-3 (PMC6791844; doi:10.1038/s41598-019-51333-3)
Supplement: Supplementary file 1 — Supplementary [file 41598_2019_51333_MOESM1_ESM.pdf]

Supplementary Information

TNFα-signal and cAMP-mediated signals oppositely regulate melanoma-associated ganglioside GD3 synthase gene in human melanocytes

Rika Takeuchi, Mariko Kambe, Maiko Miyata, Upul Jeyadevan, Orie Tajima, Koichi Furukawa, Keiko Furukawa*

Department of Biomedical Sciences, Chubu University College of Life and Health Sciences, Matsumoto 1200, Kasugai, Aichi 487-8501, Japan

*Corresponding author: Keiko Furukawa: Tel.: +81-568-51-6704;
Fax: +81-568-51-6704; E-mail: keikofu@isc.chubu.ac.jp

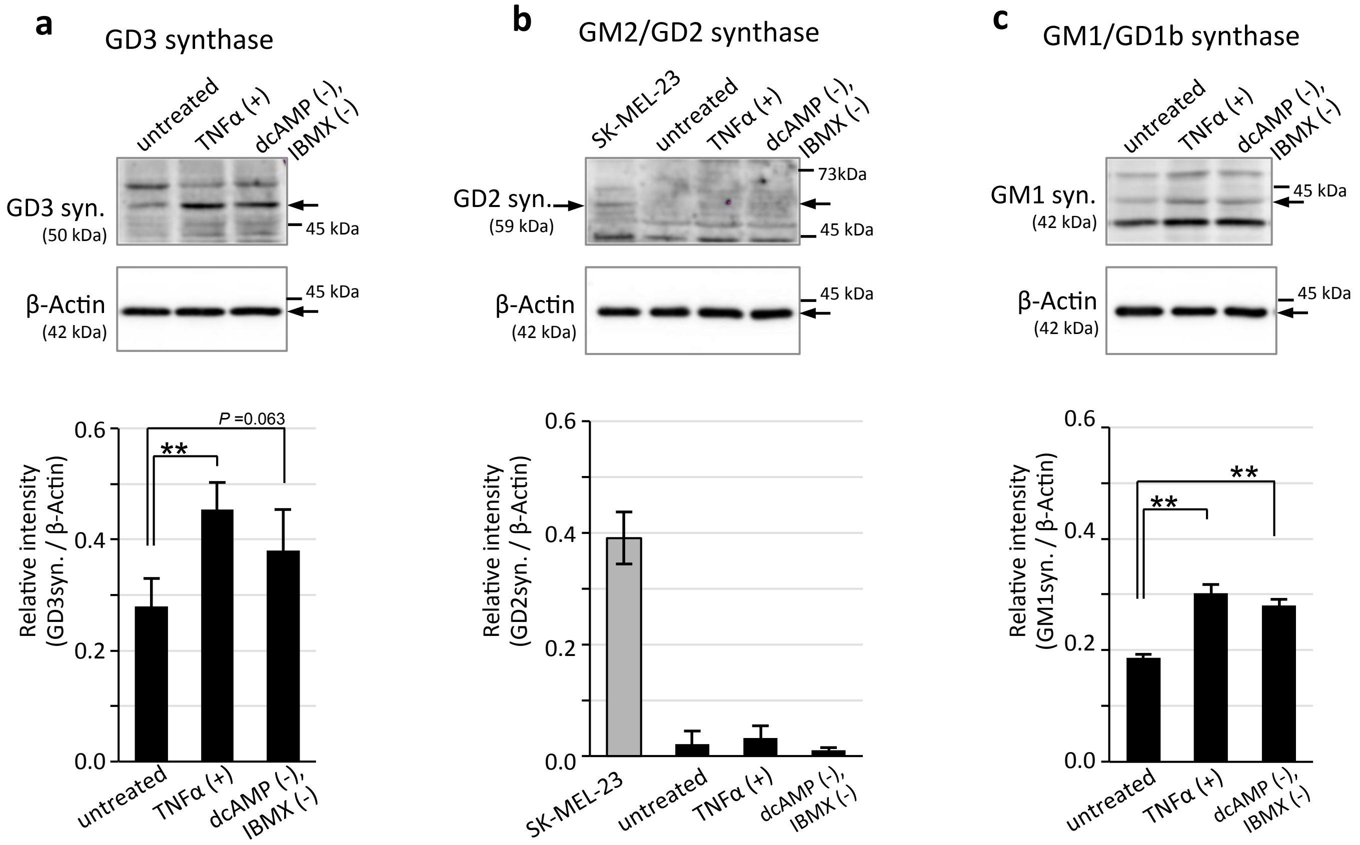

Supplementary Figure S1. GD3 synthase was increased after adding TNFα or removing dcAMP and IBMX in melanocytes.

Melanocytes were cultured in F10-A medium (containing 1m M dcAMP and 0.1 mM IBMX) for 4 days, then cultured in F10-A medium adding TNFα (10 ng/mL) for 8 h, or F10-A medium deleted of dcAMP and IBMX for 8h. Then, cell lysates were prepared, and western immunoblotting using anti-GD3 synthase, anti-GM2/GD2 synthase, or anti-GM1/GD1b synthase was performed. (a) GD3 synthase was increased after adding TNFα (10 ng/mL) or removing dcAMP and IBMX in melanocytes. (b) GM2/GD2 synthase was not detected after any treatment of melanocytes. A melanoma cell line, SK-MEL-23 (left end) was used as a positive control for GM2/GD2 synthase. (c) GM1/GD1b synthase was slightly increased after these treatments, although mRNA levels were not increased clearly (Fig. 2a, b) . Band intensities of individual synthases were measured and presented after correction by those of β-actin. Data represent means ± s.d. (n=3-4). Statistical analysis was performed by the two-tailed Student's t-test (**, *P* <0.01).

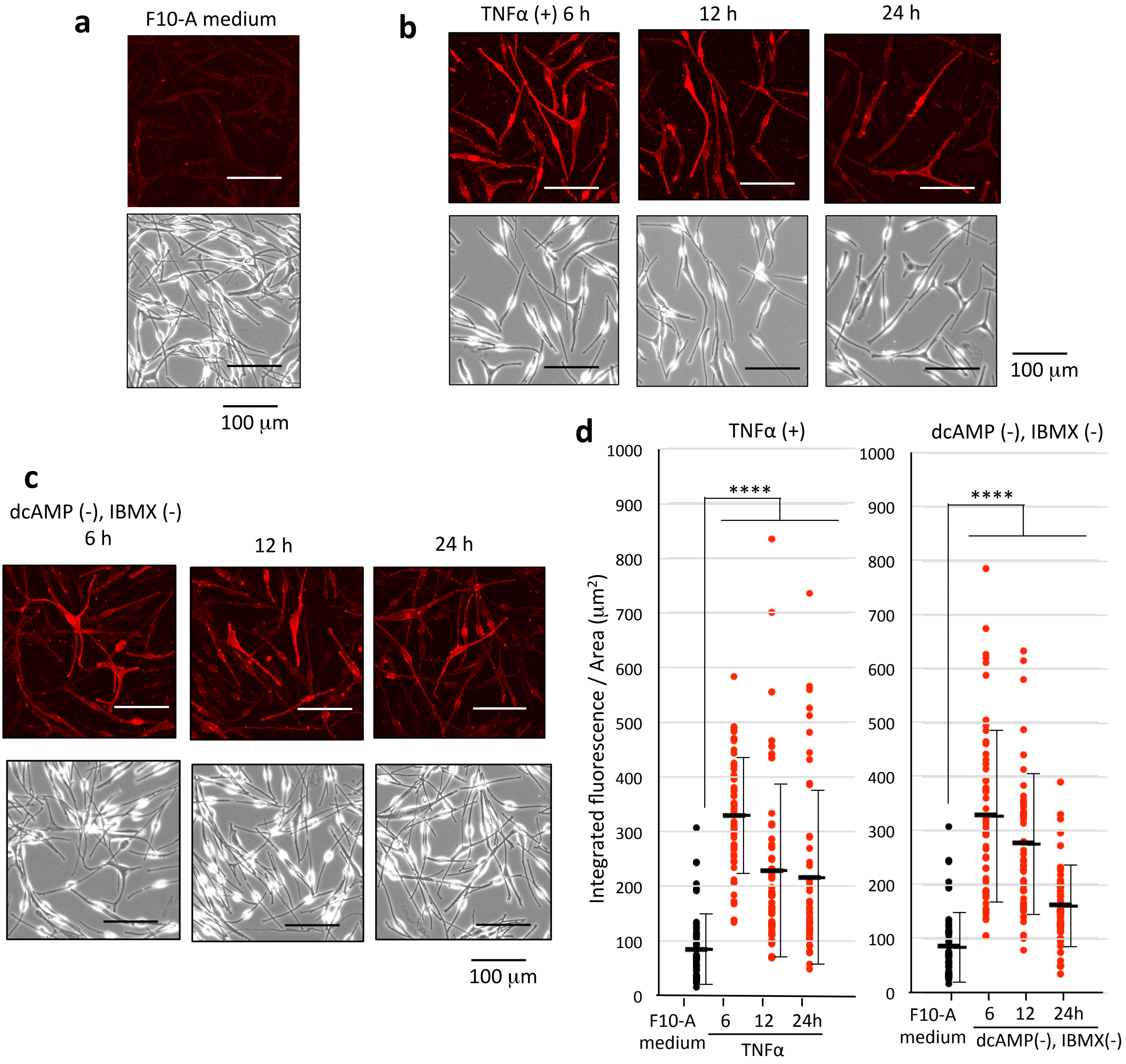

Supplementary Figure S2. Immunocytostaining of GD3 expressed after TNFα treatment or removal of dcAMP and IBMX from culture medium in melanocytes.

Results of immunocytostaining with anti-GD3 antibody (R24) and Alexa Fluor 594-anti-mouse IgG were shown. Stained GD3 was observed using a confocal microscope (*upper*), and in bright field (*lower*). (a) Melanocytes were cultured in F10-A medium. (b) GD3 expression was analyzed at 6, 12, and 24 h after the addition of TNFα (10 ng/mL) to F10-A medium. (c) GD3 expression was analyzed at 6, 12, and 24 h after the removal of dcAMP and IBMX from the F10-A medium.

(d) Quantification of fluorescent intensity for stained GD3 on melanocytes were performed. Melanocytes were cultured in F10-A medium for 24 h, then in F10-A medium with TNFα, or F10-A medium without dcAMP and IBMX for 6, 12, and 24 h. The fluorescence intensity in each cell was measured under a confocal microscope FLUOVIEW Fv10^{TM} (OLYMPUS) and presented as integrated fluorescence emission per area of single cell (µm^2). Similar results were obtained in three experiments (n=45-56 for each experiment), and representative results are shown. Data represent means ± s.d. (n=45-56). Statistical analysis was performed by the two-tailed Student's t-test (****, *P* <0.0001).

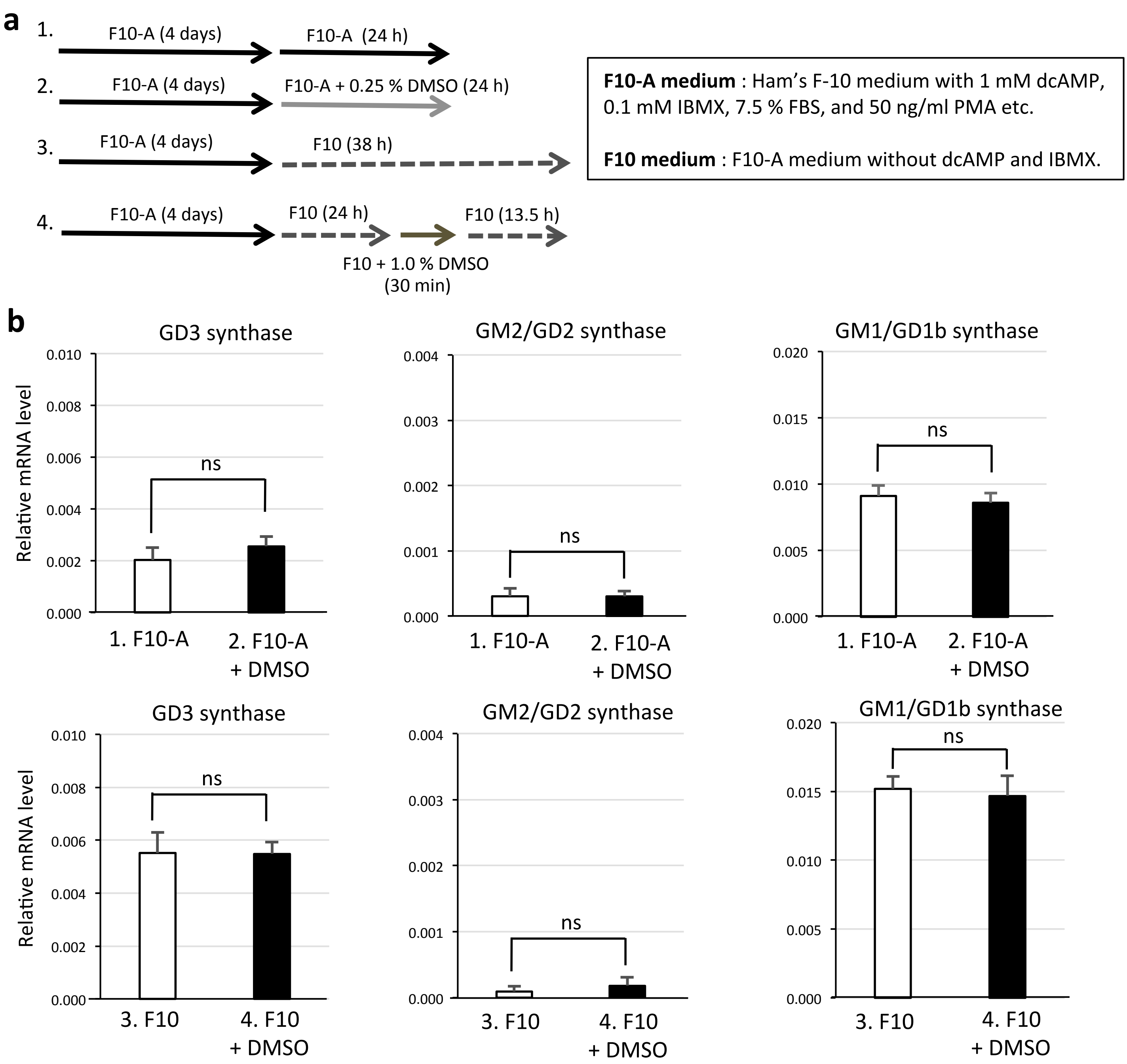

Supplementary Figure S3. Expression levels of glycosyltransferase genes in melanocytes cultured with DMSO alone were not different from those in melanocytes cultured without DMSO.

(a) Melanocytes were cultured according to protocols 1~4. Melanocytes were cultured in F10-A medium for 4 days. Then, 1. melanocytes were cultured in F10-A medium for 24 h, 2. cultured in F10-A medium with 0.25 % DMSO for 24 h, 3. cultured in F10 medium for 36 h, and 4. cultured in F10 medium for 24 h, then in F10 medium with 1.0% DMSO for 30 min, after that in F10 medium without DMSO for 13.5 h. DMSO was added up to 0.25 % of concentration to dissolve reagents (H89 or WDL) in F10-A medium. DMSO was used at the concentration of 1.0 % to dissolve α-MSH in F10 medium. (b) After treatment of melanocytes with or without DMSO in (a), mRNA expression levels of GD3 synthase, GM2/GD2 synthase, and GM1/GD1b synthase were analyzed by qRT-PCR. mRNA expression levels of these glycosyltransferases were normalized by that of human GAPDH gene. Data represent means ± s.d. (n=3-4). Statistical analysis was performed by the two-tailed Student's t-test. ns = not significant. Expression levels of glycosyltransferase genes in melanocytes cultured in F10-A or F10 medium with DMSO were almost same as those in melanocytes cultured without DMSO.

a

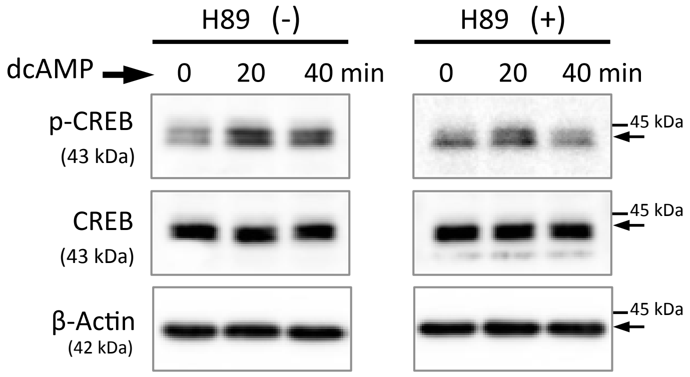

b

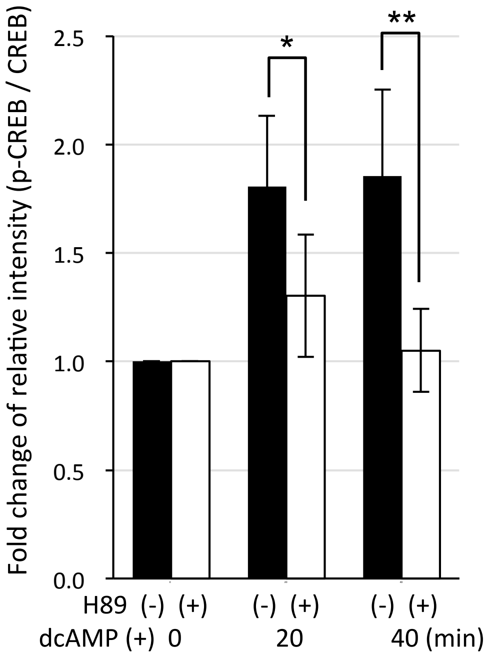

Supplementary Figure S4. Phosphorylation levels of CREB were decreased by treatment with a PKA inhibitor, H89 in melanocytes .

(a) Effects of a PKA inhibitor, H89, on phosphorylation of CREB was examined in melanocytes. Melanocytes were cultured in F10-A medium for 4 days and in F10-A medium deleted of dcAMP and IBMX for 17 h. Then, cells were cultured with or without H89 (10 mM) for 1 h, and stimulated with dcAMP (1 mM) for 0, 20, 40 min. Subsequently, cell lysates were prepared and phosphorylation of CREB was examined by western immunoblotting using anti-CREB mAb and anti-phospho-CREB (Ser133) mAb. (b) Band intensities of p-CREB were measured and presented after correction by those of CREB, then fold changes were shown as bar graphs. Data represent means ± s.d. (n=6). Statistical analysis was performed by the two-tailed Student's t-test (*, *P* <0.05; **, *P* <0.01).

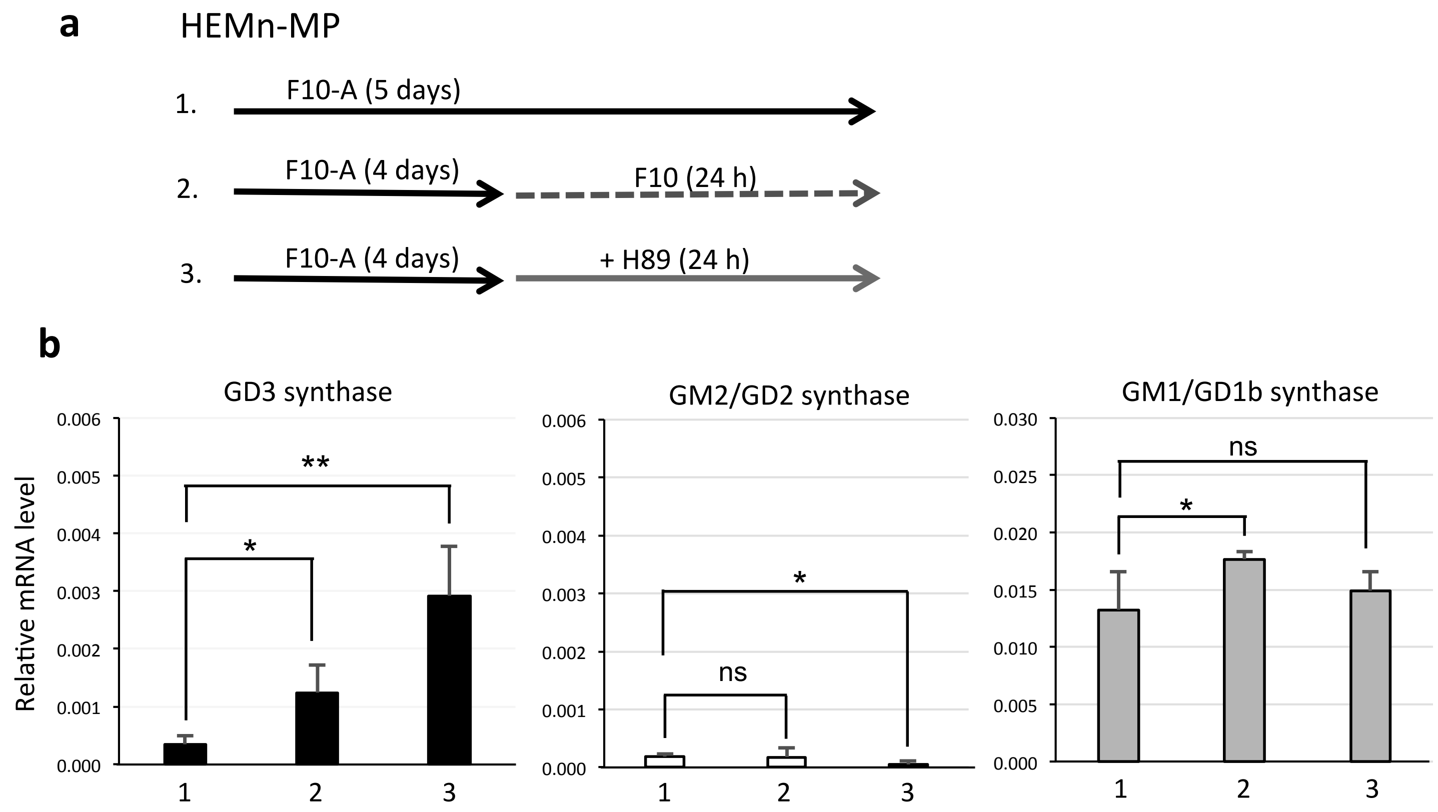

Supplementary Figure S5. Expression of GD3 synthase gene was suppressed via cAMP-dependent protein kinase (PKA) signaling pathway in melanocytes (HEMn-MP).

(a) Melanocytes (HEMn-MP) were cultured according to protocols 1~3. Melanocytes were cultured in F10-A medium for 4 days. Then, 1. melanocytes were cultured in F10-A medium for 24 h, 2. cultured in F10 medium (no dcAMP and IBMX) for 24 h, 3. cultured in F10-A medium with H89 (10 μM) for 24 h.

(b) Melanocytes were cultured as shown in (a), and mRNA expression levels of GD3 synthase, GM2/GD2 synthase, and GM1/GD1b synthase were analyzed by qRT-PCR. mRNA expression levels of these glycosyltransferases were normalized by that of human GAPDH gene. Data represent means ± s.d. (n=3-4). Statistical analysis was performed by the two-tailed Student's t-test (*, P <0.05; **, P <0.01). ns = not significant. Expression of the GD3 synthase gene was increased after removing dcAMP and IBMX or adding the PKA inhibitor H89 (10 μM), i.e., by inhibition of PKA signaling in melanocytes.

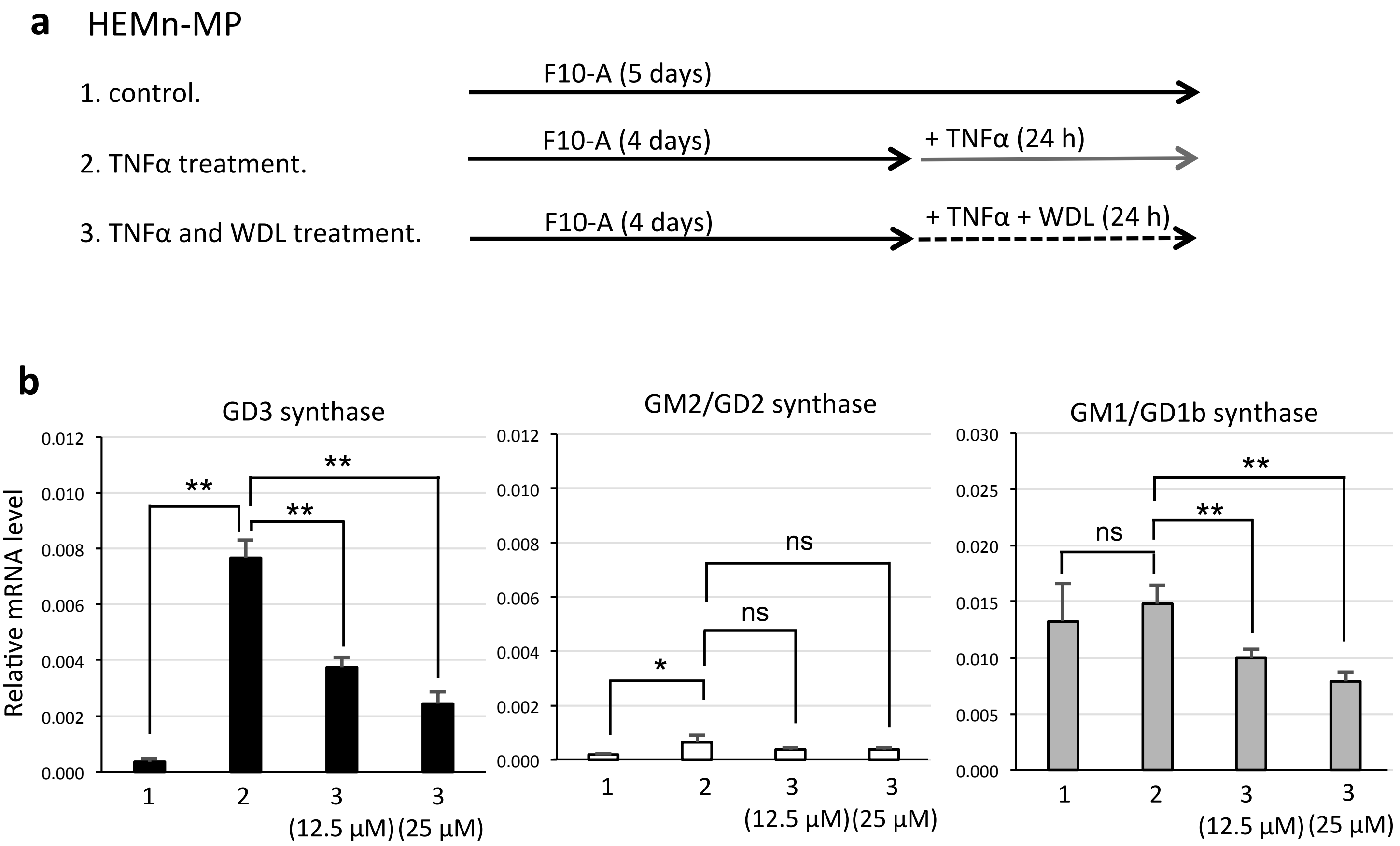

Supplementary Figure S6. TNFα induces expression of GD3 synthase gene, and inhibition of IKK results in suppression of the gene expression in melanocytes (HEMn-MP).
(**a**) Melanocytes (HEMn-MP) were cultured according to protocols 1~3. Melanocytes were cultured in F10-A medium for 4 days. Then, 1. melanocytes were cultured in F10-A medium for 24 h, 2. cultured with TNFα (10 ng/mL) for 24 h, and 3. cultured with TNFα (10 ng/mL) and an IKK inhibitor, WDL (12.5 μM or 25 μM) for 24 h. (**b**) After treatment of melanocytes with or without TNFα and WDL as in (**a**), mRNA expression levels of GD3 synthase, GM2/GD2 synthase, and GM1/GD1b synthase were analyzed by qRT-PCR. mRNA expression levels of these glycosyltransferases were normalized by that of human GAPDH gene. Data represent means ± s.d. (n=3-4). Statistical analysis was performed by the two-tailed Student's t-test (*, P <0.05; **, P <0.01). ns = not significant. Expression of the GD3 synthase gene was increased by TNFα treatment, and was suppressed by an IKK inhibitor, WDL, in melanocytes.

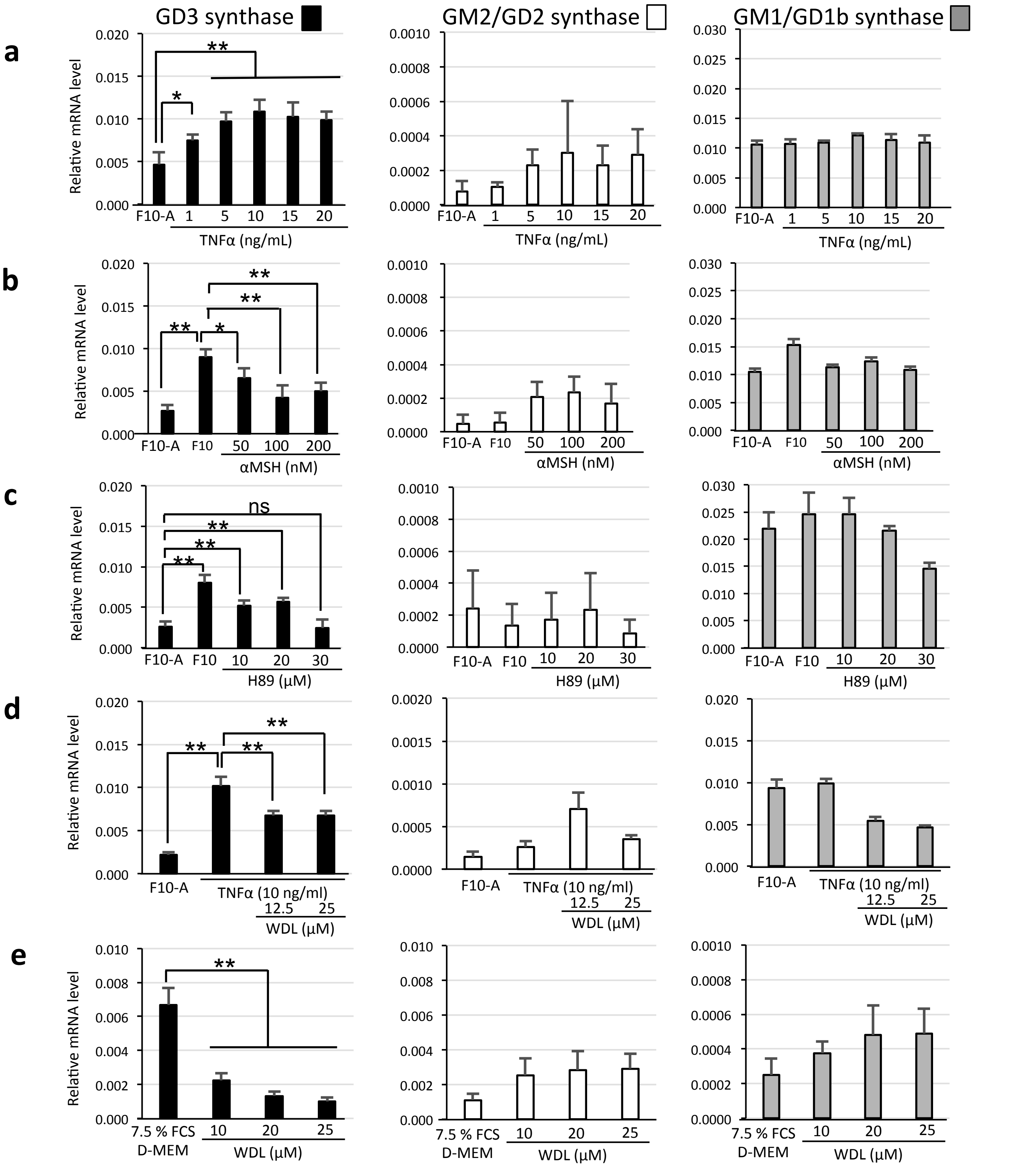

Supplementary Figure S7. Optimal concentrations of TNFα, αMSH, H89, and WDL were determined by titration. mRNA expression levels of GD3 synthase (■), GM2/GD2 synthase (□), and GM1/GD1b synthase (■) in melanocytes (HEMn-LP) and melanoma cell line (SK-MEL-28) were analyzed by qRT-PCR after treatment as follows. (a) Melanocytes were treated with TNFα (1, 5, 10, 15, or 20 ng/mL) for 24 h. Ten ng/mL of TNFα was used thereafter for experiments. (b) Melanocytes were treated with αMSH (50, 100, or 200 nM) for 30 min. A hundred nM of αMSH was used. (c) Melanocytes were treated with H89 (10, 20, or 30 μM) for 24 h. Ten μM of H89 was used. (d) Melanocytes were treated with TNFα (10 ng/mL) and WDL (12.5 or 25 μM) for 24 h. Twelve point five or 25 μM WDL was used. (e) Melanoma cell line (SK-MEL-28) was treated with WDL (10, 20, or 25 μM) for 24 h. Twenty μM WDL was used. mRNA expression levels of these glycosyltransferases were normalized by that of human GAPDH gene. Data represent means ± s.d. (n=4). Statistical analysis was performed by the two-tailed Student's t-test (*, P <0.05; **, P <0.01). ns = not significant.
